# The Prognostic and Immune Significance of *CILP2* in Pan-Cancer and Its Relationship with the Progression of Pancreatic Cancer

**DOI:** 10.3390/cancers15245842

**Published:** 2023-12-14

**Authors:** Danxi Liu, Cong He, Zonglin Liu, Licheng Xu, Jiacheng Li, Zhongjie Zhao, Xuewei Hu, Hua Chen, Bei Sun, Yongwei Wang

**Affiliations:** 1 Department of Pancreatic and Biliary Surgery, The First Affiliated Hospital of Harbin Medical University, Harbin 150001, China; 202101181@hrbmu.edu.cn (D.L.); zhaozhongjie@hrbmu.edu.cn (Z.Z.);; 2 Key Laboratory of Hepatosplenic Surgery, Ministry of Education, The First Affiliated Hospital of Harbin Medical University, Harbin 150001, China; 3 Department of Otorhinolaryngology, Head and Neck Surgery, The Second Affiliated Hospital of Harbin Medical University, Harbin 150001, China; 4 The Key Laboratory of Myocardial Ischemia, Ministry of Education, The Second Affiliated Hospital of Harbin Medical University, Harbin 150001, China

**Keywords:** cartilage intermediate layer protein 2, pan-cancer, clinical prognostic value, immunological characteristics, pancreatic cancer

## Abstract

**Simple Summary:**

In recent years, the incidence of cancer has been steadily increasing, necessitating a more urgent and in-depth understanding and exploration of this disease. During the process of literature review, we have discovered that the novel protein-coding gene, cartilage intermediate layer protein 2, has been partially associated with colorectal cancer in several studies. However, its role in other cancers remains elusive. Therefore, this study aims to explore the function and mechanism of cartilage intermediate layer protein 2 in pan-cancer. Our findings will enhance the understanding of the role of cartilage intermediate layer protein 2 in pan-cancer tumorigenesis and progression, especially in pancreatic cancer. This research could potentially establish cartilage intermediate layer protein 2 as a promising prognostic biomarker and immunotherapy target for cancers. The elucidation of its significance in pan-cancer will contribute to the development of more effective therapeutic strategies.

**Abstract:**

Cartilage intermediate layer protein 2 (*CILP2*) facilitates interactions between matrix components in cartilage and has emerged as a potential prognostic biomarker for cancer. This study aimed to investigate the function and mechanisms of *CILP2* in pan-cancer. We evaluated the pan-cancer expression, methylation, and mutation data of *CILP2* for its clinical prognostic value. Additionally, we explored the immunological characteristics of *CILP2* in pan-cancer and then focused specifically on pancreatic ductal adenocarcinoma (PAAD). The subtype analysis of PAAD identified subtype-specific expression and immunological characteristics. Finally, in vitro and in vivo experiments assessed the impact of *CILP2* on pancreatic cancer progression. *CILP2* exhibited high expression in most malignancies, with significant heterogeneity in epigenetic modifications across multiple cancer types. The abnormal methylation and copy number variations in *CILP2* were correlated with poor prognoses. Upregulated *CILP2* was associated with *TGFB*/*TGFBR1* and more malignant subtypes. *CILP2* exhibited a negative correlation with immune checkpoints in PAAD, suggesting potential for immunotherapy. *CILP2* activated the AKT pathway, and it increased proliferation, invasion, migration, and epithelial–mesenchymal transition (EMT) in pancreatic cancer. We demonstrated that *CILP2* significantly contributes to pancreatic cancer progression. It serves as a prognostic biomarker and a potential target for immunotherapy.

## 1. Background

Cancer continues to impose a substantial strain on global public health, with its impacts extending to millions of individuals across the world [1]. It is estimated that the number of cancer cases worldwide will reach 28.4 million by 2040, which represents a significant increase of 47% compared to the 2020 figures [2]. This escalating trend underscores the urgency for a more profound understanding and effective management of cancer. Over recent decades, breakthroughs in biology have propelled oncology into the era of molecular medicine, which has thoroughly revolutionized our insights into cancer [3]. This leap has allowed us to dissect the disease at the molecular level, thereby broadening our comprehension of cancer development. Meanwhile, these specific molecules, such as *KRAS*, *P53*, and so on, may act as biomarkers, therapeutic targets, or prognostic indicators for the management of cancer [4]. Traditionally, the investigation of these molecules was limited to specific cancer types. However, rapid advancements in high-throughput sequencing and multiomics technologies have led to an explosion in biological data, enabling the characterization of single genes on a pan-cancer scale [5].

Cartilage intermediate layer protein 2 (*CILP2*) is a novel protein-coding gene that encodes a secretory protein located in the extracellular matrix (ECM). Notably, compared to its paralog, *CILP1*, *CILP2* appears to be more abundant in the deeper middle region of the articular cartilage, playing roles in cartilage scaffolding and dinucleotide phosphatase activity [6,7]. Recent studies have identified *CILP2* as a prognostic biomarker associated with immune infiltration in colorectal cancer [8,9]. However, the significance of *CILP2* in other cancers remains largely unexplored.

In this study, we conducted an analysis of the expression level, methylation, copy number variation (CNV), prognostic role, and immune infiltration of *CILP2* in pan-cancer. Considering the high expression level and prognostic significance of *CILP2* in pancreatic ductal adenocarcinoma (PDAC), we specifically investigated its role in PDAC. The additional insight was that high expression levels of *CILP2* were significantly associated with more aggressive subtypes in PDAC. The subsequent enrichment analysis and rescue experiments suggested that *CILP2* could mediate the proliferation, invasion, and metastasis of PDAC through the AKT pathway, which was validated through in vivo and in vitro experiments. Considering the latent immunogenic role of *CILP2*, we also tested the effect of a combination of immune checkpoint blockers and the genetic ablation of *CILP2* on PDAC. Our study highlights the potential carcinogenic role of *CILP2* and identifies it as a prospective and promising therapeutic target for cancer and a marker of immune infiltration and poor prognosis.

## 2. Methods

### 2.1. Data Collection and Preparation

The RNA sequencing (RNA-seq) data, whole genome/exome sequencing (WGS/WES) data, and methylation data for 33 cancers were obtained from The Cancer Genome Atlas (TCGA) database (https://portal.gdc.cancer.gov/, accessed on 15 February 2023). The transcriptomic data for normal tissues was obtained from the Genotype-Tissue Expression (GTEx) database (https://www.gtexportal.org/home/, accessed on 23 February 2023). The cell line gene expression profiles for tumors were downloaded from the Cancer Cell Line Encyclopedia (CCLE) dataset (https://portals.broadinstitute.org/ccle/about, accessed on 24 February 2023) [10]. The gene expression profiles for GSE15471 and GSE102238 datasets were obtained from the Gene Expression Omnibus (GEO) database (http://www.ncbi.nlm.nih.gov/geo/, accessed on 25 February 2023). The expression profiles were normalized to the transcripts per kilobase million (TPM) format and a log2 (TPM + 0.001) transformation was conducted. For the subsequent analysis, patients were classified into low and high groups based on their median *CILP2* expression values. A set of 150 samples, referred to as the “Freeze Set”, was filtered from the TCGA-PAAD cohort based on the definition by The Cancer Genome Atlas Research Network, which was used to conduct the subsequent analysis of PDAC [11].

### 2.2. Demographic and Clinical Characteristics

We defined the demographic variables to include gender, age, race, smoking status, and alcohol consumption. The clinical variables consisted of tumor subdivision, tumor status, lymph node status, distant metastasis, pathologic stage, pathologic grade, and subtypes of PDAC as described by Moffit et al., Collisson et al., and Bailey et al. [12,13,14]. Differences in the demographic and clinical characteristics between the low and high *CILP2* expression groups were analyzed using either the Wilcoxon rank sum test or Fisher’s exact test. *CILP2* expression for the diverse clinical subgroups was analyzed using either the Student’s *t*-test or ANOVA. Cases with insufficient or missing data were excluded from the analysis.

### 2.3. Pan-Cancer Differential Expression, Genetic Abbreviations Landscape, and DNA Methylation Analysis

The TCGA and GTEx transcriptomic data were used to analyze the differential expression and DNA methylation of *CILP2* in tumor and normal tissues. TCGA samples were filtered to include only primary tumors, while samples labeled as metastatic, normal, or other conditions were excluded. The Mann–Whitney U test was applied to investigate the differential expression of *CILP2* between the tumor and normal tissues across the various types of cancers. The R4.2.0 software was used to analyze the data and generate the plots. Additionally, a pan-cancer analysis of *CILP2* genetic alterations, including mutation, structural variation, amplification, deep deletion, and multiple alterations, was performed using the cBioPortal web platform (https://www.cbioportal.org/, accessed on 25 February 2023) [15,16].

### 2.4. Survival Analysis

The TCGA clinical data containing the 33 types of cancers were included to analyze patient survival. The R packages “survival” and “survminer” were utilized to conduct the survival analysis. Samples with overall survival lengths of less than one month were excluded from the analysis. A Kaplan–Meier (KM) analysis was conducted to compare the survival rates between the low and high *CILP2* expression groups. Additionally, a univariate Cox regression analysis was used to identify the hazard ratio (HR) with 95% confidence intervals for *CILP2* in the 33 types of cancers. The cutoff for the *p*-value for significance was set to 0.05.

### 2.5. Functional Enrichment and Gene Set Variation Analyses

Enrichment analysis and a gene set variation analysis (GSVA) were utilized to identify the signal pathways and biological effects associated with the *CILP2* gene. The “clusterProfiler” package in R software (version 4.1.0) was employed to evaluate the Gene Ontology (GO), Kyoto Encyclopedia of Genes and Genomes (KEGG), and Gene Set Enrichment Analysis (GSEA) pathways [17,18], and GSVA was conducted using the “GSVA” package. The annotation database included the GO knowledge base [19], KEGG [20], the Reactome knowledge base, and 50 hallmark gene sets from the Molecular Signatures Database (MSigDB) [21]. An adjusted *p*-value of <0.05 was set as the significant threshold. Enriched pathways were ranked by adjusted *p*-value, and the top 20 pathways were depicted.

### 2.6. TME Analysis, Immune Cell Infiltration, and Immunological Characteristics

The “ESTIMATE” package was used to estimate the components of the tumor microenvironment (TME), including the stromal, immune, ESTIMATE, and tumor purity scores, across all types of cancers [22]. The “CIBERSORT” algorithm was employed to analyze the immune cell infiltrations across the different cancer types [23]. The correlations between the *CILP2* mRNA expression and the 21 immune cell subsets and genes were quantified using the Spearman correlation coefficient. All of the above analyses were based on the cancer samples and excluded the normal tissues.

### 2.7. Patients and Specimens

PDAC and adjacent non-tumor tissues were obtained from 40 patients who underwent pancreatic resection surgery at the Department of Pancreatic and Biliary Surgery (The First Affiliated Hospital of Harbin Medical University, Harbin, Heilongjiang, China) from January 2016 to December 2018. These patients were pathologically diagnosed with pancreatic ductal adenocarcinoma. After the tumor specimens were removed, the PDAC and adjacent non-tumor tissues were immediately collected and divided into two parts. One part was rapidly frozen in liquid nitrogen and stored at −80 °C while the other was fixed in 4% paraformaldehyde and embedded in paraffin. This study received ethical approval from the Research Ethics Committee of the First Affiliated Hospital of Harbin Medical University, and each patient provided informed consent before participating in the study.

### 2.8. Cell Lines

The human pancreatic cancer cell lines PANC-1 and BxPC-3 and the human pancreatic duct epithelial cells (HPDE6-C7) were purchased from the American Type Culture Collection. The SW1990 and CFPAC-1 cell lines were obtained from the Type Culture Collection of the Chinese Academy of Sciences (Shanghai, China). The mouse pancreatic cancer cell line Panc02 was purchased from Procell (Wuhan, China). The PANC-1, HPDE6-C7, SW1990, and CFPAC-1 cell lines were routinely cultured in Dulbecco’s modified Eagle’s medium (Gibco, Waltham, MA, USA) supplemented with 10% fetal bovine serum (Gibco, Waltham, MA, USA), 100 U/mL of penicillin, and 100 mg/mL of streptomycin. The BxPC-3 and Panc02 cell lines were routinely cultured in RPMI 1640 medium (Gibco, Waltham, MA, USA) supplemented with 10% fetal bovine serum (Gibco, Waltham, MA, USA), 100 U/mL of penicillin, and 100 mg/mL of streptomycin. All cells were cultured at 37 °C with 5% CO_2_.

### 2.9. Cell Transfection

The small interfering RNA (siRNA) for *CILP2* (si-CILP2) and an siRNA-negative control (si-NC) were obtained from GenePharma (Shanghai, China). To induce the overexpression of *CILP2*, the *CILP2* cDNA was cloned into a plasmid (Genechem, Shanghai, China). For the transient transfection, cells were seeded in six-well plates and transfected with either 50 nM of siRNA or 2 μg of plasmids using Lipofectamine 3000. To establish the stable transfectants with knockdown or overexpression, lentiviruses with scrambled short hairpin RNA (shRNA) for *CILP2* (sh-CILP2) and shRNA-negative controls (sh-Ctrl) were constructed in GV112 (GeneChem, Shanghai, China). Lentiviral vectors encoding the *CILP2* and empty vectors were also constructed in GV112 (GeneChem, Shanghai, China). Stable clones were selected via puromycin over 2 weeks. The efficiency of all transfections was evaluated using quantitative real-time polymerase chain reaction (qRT-PCR) assays. The target sequences of the siRNAs are listed in Table S1.

### 2.10. RNA Extraction and qRT-PCR Analysis

The total RNA was extracted and isolated from the cell lines and frozen tumor specimens using an AxyPrep Multisource Total RNA Miniprep Kit from Axygen (Corning, Suzhou, China). The first-strand cDNA was synthesized with a Rever TraAce qPCR RT Kit Master Mix with gDNA Remover (FSQ-301, Toyobo Co. Ltd. Osaka, Osaka, Japan) following the manufacturer’s instructions. The qRT-PCR analysis was performed as previously described [24]. Briefly, qRT-PCR (SYBR Green Assay, Roche Diagnostics GmbH, Indianapolis, IN, USA) was performed on a 7500 FAST Real-Time PCR System (Applied Biosystems, Foster City, CA, USA). The relative expression levels of mRNA were calculated and quantified using the 2^−ΔΔT^ method after normalizing to the expression of the endogenous control, which was *ACTB*. The primer sequences are described in Table S2 and were purchased from Comate Bioscience (Institute of Biotechnology, Jilin, China).

### 2.11. Western Blot

Proteins were extracted from the PDAC cells using RIPA buffer (Beyotime Institute of Biotechnology, Beijing, China) containing a protease inhibitor cocktail and a phosphatase inhibitor. The extracted proteins were homogenized, and the protein samples (40 µg) were separated using SDS-PAGE and then transferred onto polyvinylidene difluoride (PVDF) membranes. The membranes were blocked with 5% non-fat milk and incubated with primary antibodies at room temperature for 2 h, followed by the addition of horseradish peroxidase (HRP)-conjugated secondary antibodies at room temperature for 1 h [25]. The bands were visualized and analyzed using Image Studio Lite software (version 5.2.5). The primary antibodies used for the Western blot analysis are listed in Table S3.

### 2.12. Cell Counting Kit (CCK- 8) Assay and Colony Assay

To assess cell proliferation, a cell counting kit (CCK-8 (Dojindo, Kumamoto, Japan)) was used following the manufacturer’s protocols. Briefly, 2000 cells were seeded into 96-well plates, 10 microliters of CCK-8 solution were added to each well, and the plate was incubated for 2 h in the dark at 37 °C. At 24, 48, 72, and 96 h, absorbance was read at 450 nm using a microplate reader (BioTek, Winooski, VT, USA). For the colony formation experiment, 1000 cells transfected with pcDNA-CILP2 and si-CILP2 and the negative control were seeded into six-well plates. After two weeks, the colonies were fixed with 4% paraformaldehyde for 30 min and stained with 1% crystal violet.

### 2.13. Wound Healing Assay and Transwell Assay

To initiate wound healing assays, an artificial wound was created using a 200 µL pipette tip. PANC-1 or BxPC-3 cells were cultured in a serum-free medium and photographed at 0 and 24 h after the wounds were created. To assess the motility and invasiveness of the cells, Falcon inserts (with an 8 µm pore size) coated or uncoated with Matrigel (BD Biosciences, Franklin Lakes, NJ, USA) were used. A total of 20,000 cells (PANC-1) or 50,000 cells (BxPC-3) suspended in a serum-free medium were seeded into the upper chamber while 500 µL of the normal medium was added to the lower part of the transwell unit. After incubation for 24 h (PANC-1 without Matrigel) or 48 h (BxPC-3 or PANC-1 with Matrigel), the cells on the upper part of the membrane were removed using a cotton swab. The cells that had invaded the bottom of the membrane were fixed in 4% paraformaldehyde and stained with 1% crystal violet.

### 2.14. BALB/c Mice Orthotopic Pancreatic Cancer Model

The experimental procedure was approved by the Institutional Review Board of the First Affiliated Hospital of Harbin Medical University. Five-week-old female BALB/c nude mice were purchased from the Charles River Company (Beijing, China). Lentiviral transfection was utilized to express the different levels of *CILP2* in the PANC-1 cells and BxPC-3 cells. For the orthotopic injection, the mice were anesthetized with intraperitoneal injections of 2.5% 2,2,2-tribromoethanol (Sigma-Aldrich, Shanghai, China), and 2 × 10^5^ cells per mouse were injected into the pancreas. After five weeks, all mice were euthanized, and the sizes and weights of the tumors were recorded. Finally, all specimens were fixed in 4% formaldehyde.

### 2.15. C57BL/6 Orthotopic Pancreatic Cancer Mice Model and Reagents

Three-week-old female C57BL/6 mice were purchased from the Charles River Company (Beijing, China). Lentiviral transfection was utilized to express the different levels of *CILP2* in the PANC02 cells. For the orthotopic injection, the mice were anesthetized with intraperitoneal injections of 2.5% 2,2,2-tribromoethanol (Sigma-Aldrich, Shanghai, China), and a total of 2 × 10^5^ cells in 10μL PBS were injected slowly into the pancreas using a 30 G needle. After 2 weeks of tumor implantation, a small-molecule PD-1/PD-L1 inhibitor, BMS202 (Selleck, Houston, TX, USA), was intraperitoneally injected at a dose of 20 mg/kg for 7 consecutive days, and the same dose of PBS was injected in the non-PD-L1 treatment group. After 1 week, all mice were euthanized, and the sizes and weights of the tumors were recorded.

### 2.16. BALB/c Metastatic Pancreatic Cancer Mice Model

Five-week-old female BALB/c nude mice were purchased from the Charles River Company (Beijing, China). Lentiviral transfection was utilized to express the different levels of *CILP2* in the PANC-1 cells and BxPC-3 cells. The portal veins were exposed by midline incisions, and then a total of 2 × 10^6^ cells in 100 μL PBS were injected slowly into the portal veins using a 30 G needle. Next, compression using cotton swabs was performed at the injection sites to avoid hemorrhages. The abdominal walls were closed with sutures, and the mice were allowed to recover on warming pads. The mice were euthanized 8 weeks later, and their liver metastases were measured.

### 2.17. Immunohistochemistry (IHC) Staining

The protocol used for the immunohistochemical staining has been previously described [24]. Briefly, specimens were fixed in 10% buffered formalin for 24 h, embedded in paraffin, and sectioned into 5 mm sections. The sections were subsequently deparaffinized and rehydrated, followed by incubation in 3% hydrogen peroxide for 15 min, and then they were rinsed in water and blocked in 2.5% normal horse serum. The antibodies were incubated with the sections and captured by microscopy (40×, Nikon, Tokyo, Japan). The total number of positive pixels was counted in five fields and calculated using Image J software (version 1.53s). The antibodies used in this study are listed in Table S3.

### 2.18. Statistical Analysis

The statistical analysis was performed using SPSS 22.0 software and GraphPad Prism 8.3.0 software. The data are presented as means ± standard deviations (SDs). Pearson analysis, one-way ANOVA, and the Student’s *t*-test were used to evaluate the statistical significance, with differences considered significant when *p* < 0.05.

## 3. Results

### 3.1. Tissue-Specific Expression Patterns of CILP2 in Pan-Cancer

**Figure 1:**
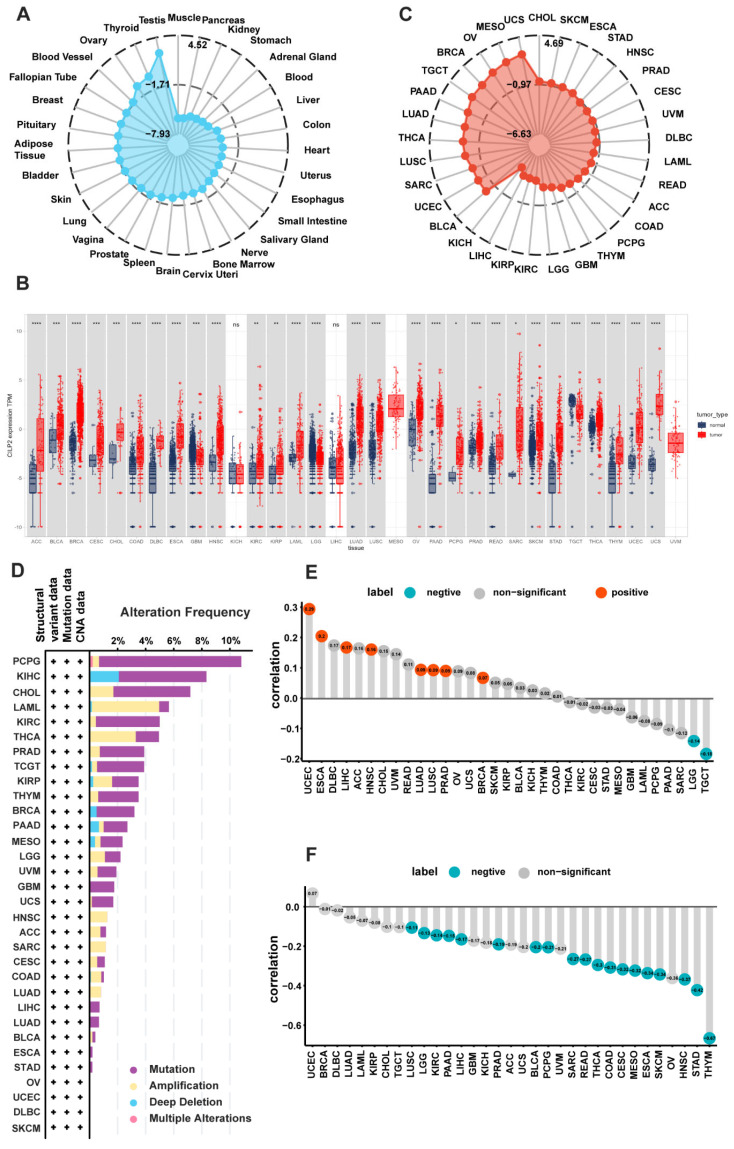
The expression of *CILP2* in pan-cancer and its genetic alteration and epigenetic variation landscape. (**A**) The expression levels of *CILP2* in normal tissues (GTEx datasets). (**B**) The differential expression levels of *CILP2* in cancer tissues and the normal counterparts from the TCGA and GTEx databases. (**C**) The expression levels of *CILP2* in the cancer cell lines (CCLE datasets). (**D**) The genetic alteration landscape of *CILP2* in pan-cancer (cBioPortal). (**E**) The correlations between the copy number variations and the mRNA expression levels of *CILP2* in pan-cancer. (**F**) The correlations between methylation and the mRNA expression levels of *CILP2* in pan-cancer. ns, no significance; *, *p* < 0.05; **, *p* < 0.01; ***, *p* < 0.001, ****, *p* < 0.0001, +, data exist.

To investigate the potential role of *CILP2*, we analyzed the mRNA expression levels of *CILP2* in the normal tissues from the GTEx database. As shown in Figure 1A, high expression levels of *CILP2* were observed in the testicle, thyroid, and ovary tissues, while low expression levels of *CILP2* were detected in the muscle, pancreas, and kidney tissues. Furthermore, the differential analysis of *CILP2* expression revealed higher expression levels in most cancers compared to the corresponding normal tissues (Figure 1B). We also analyzed the expression of *CILP2* mRNA in 1457 cell lines from 26 types of tumors in the CCLE database. Our findings revealed that *CILP2* was significantly upregulated in the cell lines from breast cancer, neuroblastomas, non-small cell lung cancer, and esophageal cancer, while it was downregulated in the cell lines from the hematopoietic system, such as multiple myeloma, chronic myeloid leukemia, and acute myeloid leukemia (Figure 1C).

### 3.2. Genetic Alterations and the Epigenetic Variation Landscape in CILP2

We investigated the genetic alteration status of the *CILP2* gene in 33 types of pan-cancer, including mutations, amplifications, deep deletions, and multiple alterations. As shown in Figure 1D, a majority of the alterations were caused by mutations, and deep deletions were less common. Skin cutaneous melanoma had the most genetic alterations in the *CILP2* gene, mainly in the form of mutations, whereas there were almost no alterations in acute myeloid leukemia, cholangiocarcinoma, renal cell carcinoma, pheochromocytoma, and paraganglioma. The copy number variation analysis revealed significantly increased copy numbers of *CILP2* in the endometrial, esophageal, and liver cancers, while they were significantly decreased in low-grade glioma and tenosynovial giant cell tumors (Figure 1E). The correlation analysis between the methylation levels and the mRNA expression levels of *CILP2* indicated that in most tumors, the methylation of *CILP2* was negatively correlated with its transcription level (Figure 1F).

### 3.3. Clinical Prognostic Significance of CILP2 in Pan-Cancer

**Figure 2:**
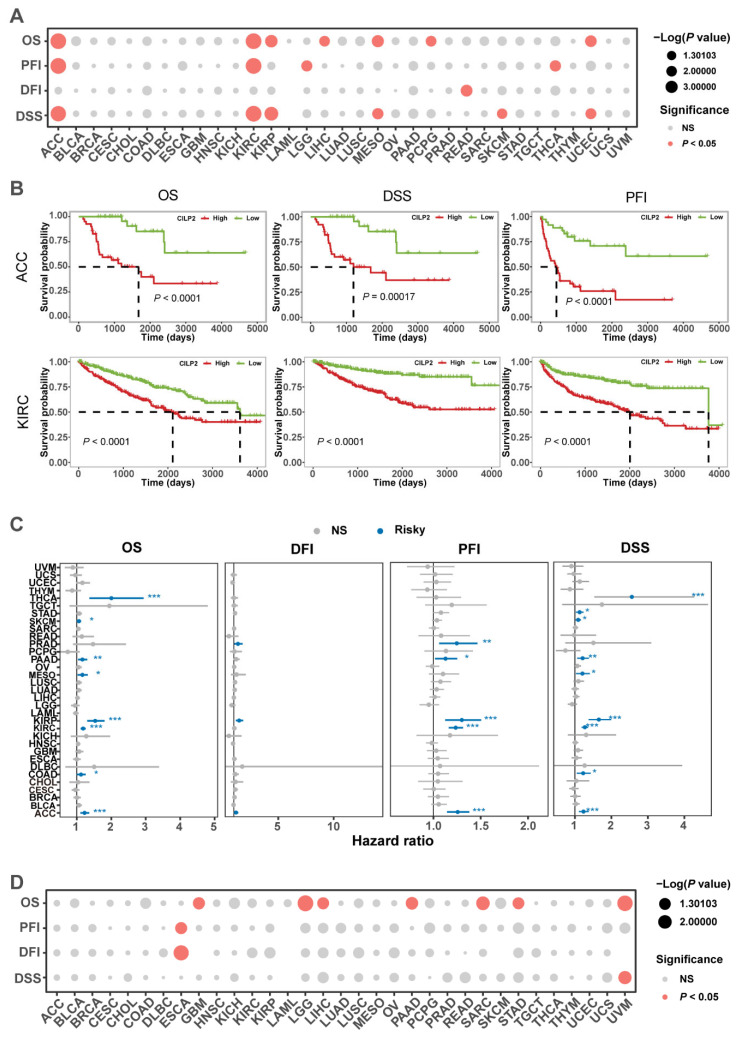
The clinical prognostic significance of *CILP2* in pan-cancer. (**A**) A summary of the correlation between *CILP2* expression and overall survival (OS), disease-specific survival (DSS), disease-free interval (DFI), and progression-free interval (PFI) in pan-cancer. (**B**) High expression levels of *CILP2* were significantly correlated with shorter OS, DSS, and DFI in adenoid cystic carcinoma (ACC) and kidney renal clear cell carcinoma (KIRC). (**C**) The prognosis value of the expression level of *CILP2* in pan-cancer through a single variate Cox regression analysis. (**D**) The prognosis value of *CILP2* low vs. high methylation levels in pan-cancer. NS, no significance; *, *p* < 0.05; **, *p* < 0.01; ***, *p* < 0.001.

To further investigate the prognostic potential of *CILP2* in cancer, we analyzed four prognostic indicators using the KM method (log-rank test) in 33 different types of cancers (Figure 2A). Our findings showed that high expression levels of *CILP2* were associated with poor prognoses in all cancer types, especially in adenoid cystic carcinoma (ACC) and kidney renal clear cell carcinoma (KIRC) (Figure 2B). To examine the contribution of *CILP2* expression level to prognosis, we conducted a univariate Cox regression analysis. The results were consistent with the log-rank test, showing a significant association between *CILP2* expression and prognosis in ACC, KIRC, and pancreatic adenocarcinoma (PAAD) (Figure 2C). Finally, to evaluate the impact of *CILP2* methylation levels on prognosis, we performed a KM analysis, which indicated that higher methylation levels of *CILP2* were associated with decreased survival rates in various types of cancers, including esophageal squamous cell carcinoma (ESCA), glioblastoma multiforme (GBM), low-grade glioma (LGG), liver hepatocellular carcinoma (LIHC), PAAD, sarcoma (SARC), stomach adenocarcinoma (STAD), and uveal melanoma (UVM) (Figure 2D and Figure S1A–J).

### 3.4. Immune Cell Infiltration Analyses of CILP2 across the Different Cancers

**Figure 3:**
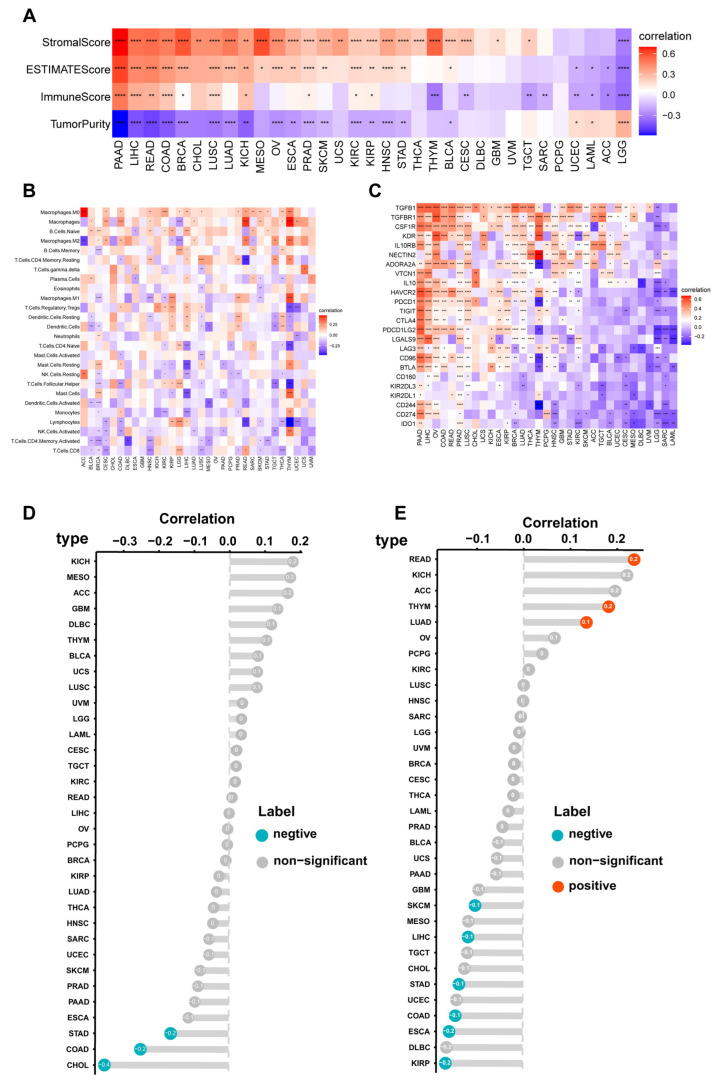
Immune cell infiltration analyses of *CILP2* across the different cancers. (**A**) The correlations between *CILP2* and the tumor purity, stromal, immune, and ESTIMATE scores in pan-cancer. (**B**) The correlation between *CILP2* and immune cell infiltration in pan-cancer. (**C**) Correlation between *CILP2* and immune suppressor genes in pan-cancer. (**D**) Correlation between *CILP2* and MSI. (**E**) Correlation between *CILP2* and TMB. *, *p* < 0.05; **, *p* < 0.01; ***, *p* < 0.001, ****, *p* < 0.0001.

Firstly, we evaluated the correlations between *CILP2* and the tumor purity score, stromal score, ESTIMATE score, and immune score in pan-cancer using the ESTIMATE algorithm (Figure 3A). The expression levels of *CILP2* showed positive correlations with the stromal score and the ESTIMATE score in most cancers, while they were negatively correlated with tumor purity and immune scores. Among them, the most significant correlation was observed in pancreatic cancer. High expression levels of *CILP2* consistently accompanied the infiltration of cancer-associated fibroblasts (CAFs) and tumor-associated macrophages (TAMs) (Figure 3B and Figure S2). Furthermore, we explored the correlations between *CILP2* expression levels and immunosuppressor genes. The outcomes varied depending on the specific tumor and gene involved (Figure 3C). In PDAC, almost all immunosuppressor genes showed positive correlations with *CILP2*. Additionally, *TGFB1*/*TGFBR1* exhibited positive correlations with *CILP2* in nearly all cancer types. Tumor mutation burden (TMB) and microsatellite instability (MSI) have been proposed as predictive biomarkers for the response to immune checkpoint blockade (ICB). Therefore, we analyzed the correlations between *CILP2*, TMB, and MSI. The results indicated that the correlations between *CILP2* and TMB varied across the different cancer types (Figure 3D). Notably, *CILP2* showed negative correlations with MSI in stomach adenocarcinoma (STAD), colon adenocarcinoma (COAD), and cholangiocarcinoma (Figure 3E).

### 3.5. CILP2 was Highly Expressed in PDAC and was Associated with Subtypes with Poorer Survival

**Figure 4:**
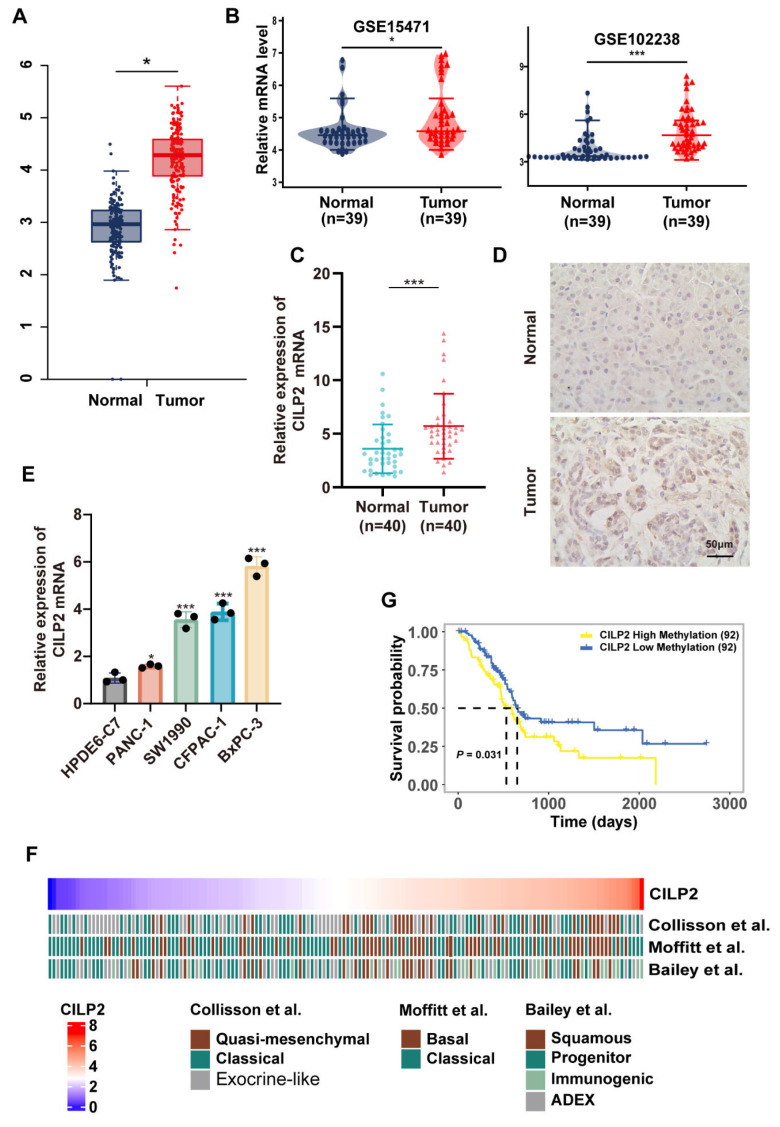
*CILP2* was highly expressed in PDAC and showed relevance to the worse subtypes and poorer survival. The differential expression levels of *CILP2* in the PDAC and normal samples: (**A**) TCGA vs. GTEx and (**B**) the GSE15471 and GSE102238 datasets. (**C**) The relative expression levels of *CILP2* in the paired pancreatic cancer tissues and the normal pancreatic tissues were detected using qRT-PCR (n = 40). (**D**) The IHC staining of *CILP2* in the paired pancreatic cancer tissues and the normal pancreatic tissues (n = 40). (**E**) The relative expression levels of *CILP2* in the pancreatic cancer cell lines and the pancreatic duct epithelial cells as detected by qRT-PCR. (**F**) The high expression levels of *CILP2* were relevant to the worse subtypes of PDAC (quasi-mesenchymal in Collisson et al., basal in Moffitt et al., and squamous in Bailey et al.) [13,14,26]. (**G**) The high methylation levels of *CILP2* were significantly correlated with shorter OS in PDAC. *, *p* < 0.05; ***, *p* < 0.001.

Based on the previous univariate COX prognostic analysis, TME analysis, and immune infiltration analysis conducted within the pan-cancer scope, all of which demonstrated the value of *CILP2* in PAAD, we further explored the expression profile of *CILP2* in PDAC (the most common and typical type of pancreatic cancer). Both the TCGA-PDAC cohort (Figure 4A) and multiple GSE datasets (Figure 4B) revealed high expression levels of *CILP2* in pancreatic cancer tissues compared to normal adjacent tissues. The results of the qRT-PCR analysis showed the significant upregulation of *CILP2* in pancreatic cancer tissues compared to their corresponding adjacent non-tumor tissues (Figure 4C). These findings were consistent with the results obtained from the IHC analysis of the 40 paired human pancreatic cancer samples (Figure 4D). Additionally, the *CILP2* mRNA levels were significantly increased in four pancreatic cancer cell lines (Figure 4E), including PANC-1, BxPC-3, SW1990, and CFPAC-1, when compared to the HPDE6-C7 line. The baseline table of clinical features also revealed differences in the *CILP2* mRNA expression levels among the different subtypes of PDAC (Table 1), though other clinical pathological features did not show any differences (Table S4). Specifically, patients with high expression levels of *CILP2* were more likely to exhibit quasi-mesenchymal/basal/squamous subtypes (Figure 4F), which are typically associated with malignant biological behaviors and poorer prognoses [26]. This finding might also explain the negative correlation between *CILP2* and patient prognosis, as was observed in the survival analysis. Furthermore, a high level of methylation in *CILP2* was correlated with shorter overall survival in PDAC (Figure 4G).

### 3.6. CILP2 Promoted the Proliferation, Migration, and Invasion of PDAC Cell Lines via the AKT Pathway

**Figure 5:**
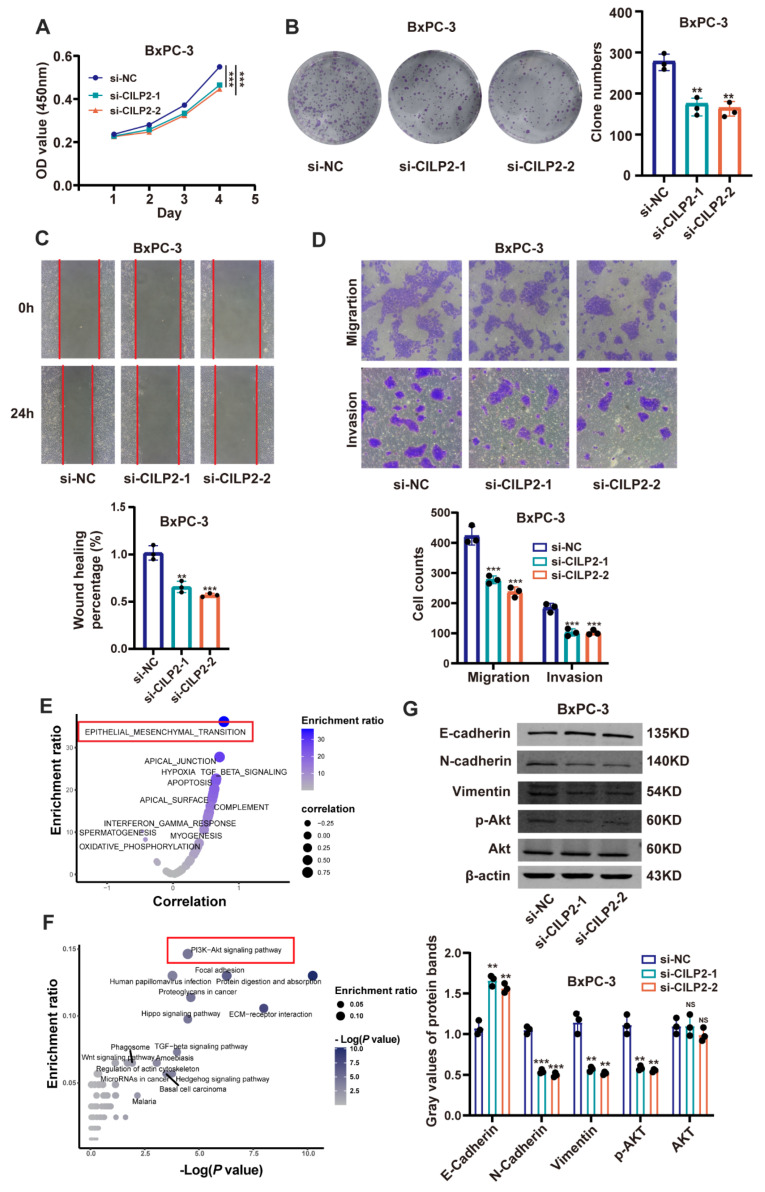
*CILP2* promoted the proliferation, migration, and invasion of the PDAC cell lines. (**A**,**B**) show the effects of *CILP2* knockdown on the proliferation of the BxPC-3 cells as measured by the CCK-8 assay and colony formation assay, respectively. (**C**,**D**) show the effects of *CILP2* knockdown on the migration and invasion of the BxPC-3 cells as measured by the transwell and wound healing assays. (**E**) The gene set variation analysis of *CILP2* in pancreatic cancer. (**F**) The KEGG enrichment analysis of *CILP2* in pancreatic cancer. (**G**) The effects of the *CILP2* knockdown on EMT and the PI3K-AKT pathway in BxPC-3 cells as measured by Western blot. Original western blots are presented in File S1. *p* < 0.05; **, *p* < 0.01; ***, *p* < 0.001.

To investigate the function and molecular mechanism of *CILP2* in pancreatic cancer cells, we generated three siRNAs to silence endogenous *CILP2* expression in the *CILP2*-high cell line BxPC-3. *CILP2* was effectively downregulated by si-CILP2-1 and si-CILP2-2. Therefore, we selected si-CILP2-1 and si-CILP2-2 for further experiments (Figure S3A). Additionally, we constructed overexpression plasmids to induce the expression of *CILP2* and transfected them into the *CILP2*-low cell line PANC-1 (Figure S3B). To explore the effect of *CILP2* on pancreatic cancer cell proliferation, we performed CCK-8 and colony formation assays. The results revealed that the knockdown of *CILP2* significantly inhibited the proliferation of BxPC-3 compared to the control group (Figure 5A,B). In contrast, the overexpression of *CILP2* significantly promoted the proliferation of PANC-1 (Figure S3C,D). Subsequently, the transwell and wound healing assays were conducted to evaluate the effect of *CILP2* on the migratory and invasive properties of pancreatic cancer cells. The results demonstrated that the knockdown of *CILP2* reduced the migration and invasion of BxPC-3 (Figure 5C,D), whereas the upregulation of *CILP2* in PANC-1 accelerated cell migration and invasion (Figure S3E,F). The invasion and metastasis of malignant tumors are largely attributed to the critical role played by the epithelial–mesenchymal transition (EMT) in this process. The gene set variation analysis (GSVA) suggested a close relationship between *CILP2* and EMT in pancreatic cancer (Figure 5E). Further, the KEGG enrichment analysis indicated a significant association between *CILP2* and the PI3K-AKT pathway in pancreatic cancer (Figure 5F). Therefore, we further explored the effect of *CILP2* on the EMT process and the PI3K-AKT pathway. The Western blot showed that the knockdown of *CILP2* decreased the expression of the mesenchymal marker N-cadherin and vimentin and increased the expression of the epithelial marker E-cadherin. On the contrary, the opposite results were observed in the PANC-1 cells with *CILP2* upregulation. Moreover, the Western blot revealed that the knockdown of *CILP2* inhibited the phosphorylation of AKT while the overexpression of *CILP2* produced the opposite result (Figure 5G and Figure S3G).

**Figure 6:**
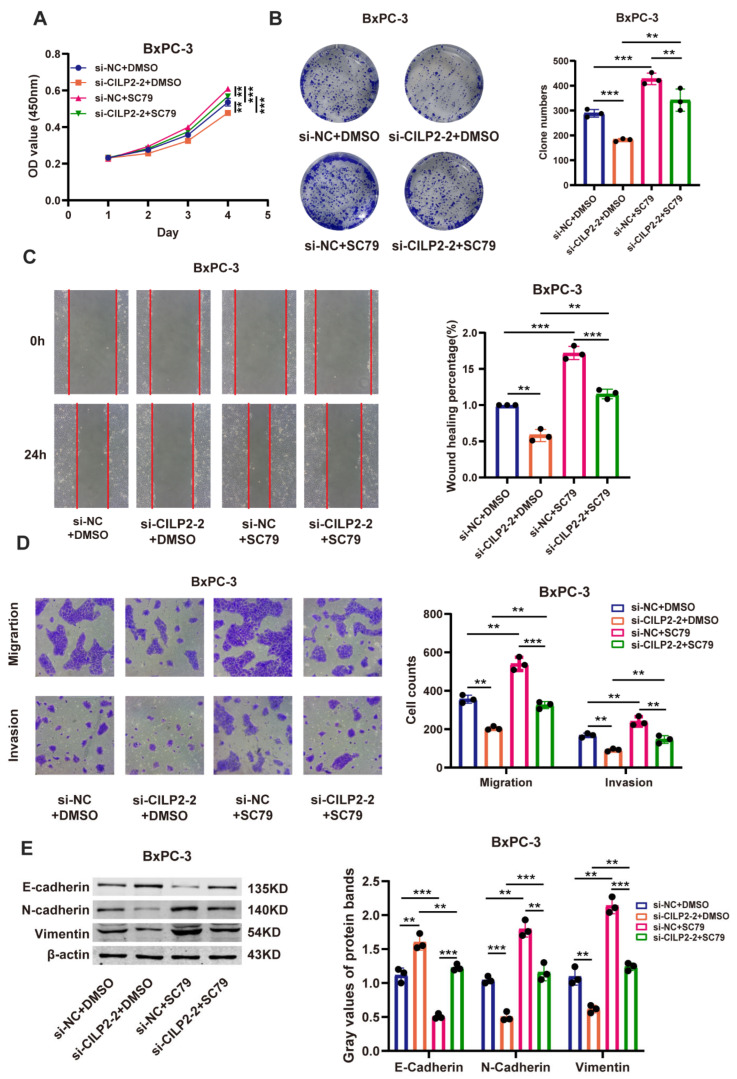
*CILP2* promoted the proliferation, migration, and invasion of the PDAC cell lines via the PI3K-AKT pathway. (**A**,**B**) show the CCK-8 and colony formation assays, respectively, used to examine cell proliferation in the BxPC-3 cells. The cells were transfected with si-CILP2 and treated with SC79 or a corresponding negative control. (**C**,**D**) show the transwell and wound healing assays, respectively, used to examine the cell migration and invasion of the BxPC-3 cells. The cells were transfected with si-CILP2 and SC79 or a corresponding negative control. (**E**) The Western blot assay used to examine the expression of EMT in the BxPC-3 cells. The cells were transfected with si-CILP2 and treated with SC79 or a corresponding negative control. Original western blots are presented in File S1. **, *p* < 0.01; ***, *p* < 0.001.

To determine whether the AKT pathway signaling participates in the oncogenic functions of *CILP2*, rescue assays were conducted to examine the effects of *CILP2* and AKT pathway signaling on cell proliferation, migration, invasion, and EMT in PDAC cells. The results indicated that SC79, an agonist of AKT pathway signaling, partially rescued the promotive effects of the *CILP2* knockdown (Figure 6A–E). Conversely, LY294002, an inhibitor of AKT pathway signaling, exhibited the opposite effect on *CILP2* overexpression (Figure S4A–E). Overall, our results revealed that *CILP2* might act as an oncogenic factor in regulation through the AKT pathway signaling, and it might play an oncogenic role in pancreatic cancer.

### 3.7. The Knockdown of CILP2 Inhibited the Progression of Pancreatic Cancer and Synergized with ICIs In Vivo

**Figure 7:**
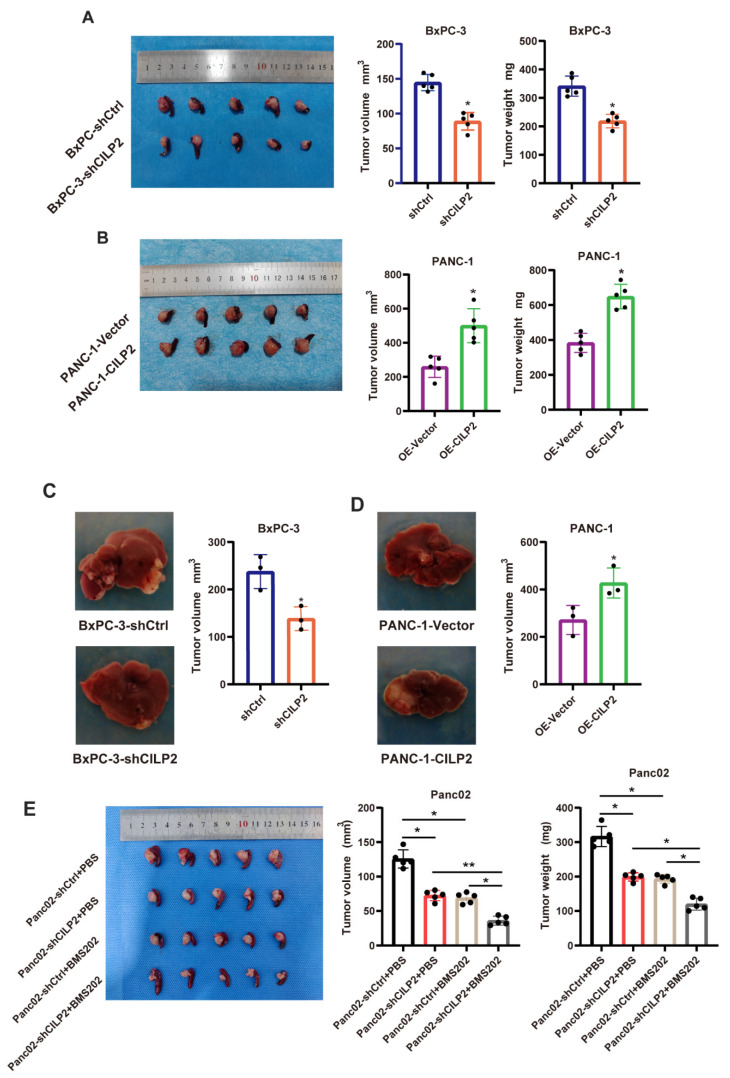
The knockdown of *CILP2* inhibited the progression of pancreatic cancer in vivo. (**A**) The tumor volumes and weights of the orthotopic tumors in the BxPC-3-shCtrl and BxPC-3-shCILP2 groups. (**B**) The tumor volumes and weights of the orthotopic tumors in the PANC-1-Vector and PANC-1-CILP2 groups. (**C**) The tumor volumes of the liver metastatic tumors in the BxPC-3-shCtrl and BxPC-3-shCILP2 groups. (**D**) The tumor volumes of the liver metastatic tumors in the PANC-1-Vector and PANC-1-CILP2 groups. (**E**) The tumor volumes and weights of the Panc02-shCILP2 orthotopic tumors in the mice treated with the vehicle control (Ctrl) or BMS202 (20 mg/kg). *, *p* < 0.05; **, *p* < 0.01.

To investigate the role of *CILP2* in pancreatic cancer progression in vivo, we developed orthotopic and liver metastasis pancreatic cancer models using stably transfected cell lines (PANC-1-Vector, PANC-1-CILP2, BxPC-3-shCtrl, and BxPC-3-shCILP2). As demonstrated in Figure 7A, compared to the control groups, the *CILP2* knockdown group exhibited lower average tumor volumes and weights while high expression levels of *CILP2* resulted in larger tumor volumes and heavier tumor weights in the orthotopic pancreatic cancer model (Figure 7B). Similarly, the volumes of the liver metastatic nodules in the BxPC-3-shCILP2 group were much lower than those in the BxPC-3-shCtrl group (Figure 7C), while the overexpression of *CILP2* showed the opposite results (Figure 7D), which were consistent with the in vitro phenotypes. Furthermore, the IHC analysis showed that the decreased expression of *CILP2* led to decreased levels of Ki-67, N-cadherin, and vimentin, as well as increased levels of E-cadherin in the orthotopic pancreatic cancer model. Conversely, the opposite effects were observed in the *CILP2* overexpression group (Figure S5A).

We also investigated the effect of the CILP2 knockdown combined with ICIs (immune checkpoint inhibitors) using an immunocompetent mouse model. First, we generated three murine siRNAs to silence endogenous CILP2 expression in the mouse pancreatic cancer cell line Panc02, and the downregulation of CILP2 was most significant by si-CILP2-1-mouse (Figure S5B). Based on the siRNAs sequence, we constructed corresponding shRNAs. After establishing stably transfected Panc02 cell lines (Panc02-shCtrl and Panc02-shCILP2), an orthotopic xenograft model was generated by injecting CILP2 knockdown cell lines, and these were subsequently treated with or without BMS202, a small-molecule PD-1/PD-L1 inhibitor. The CILP2 knockdown group and the anti-PD-L1 therapy-only group showed tumor regression compared with the control group, respectively. Further, compared with the CILP2 knockdown group and the anti-PD-L1 therapy-only group, there was significant tumor regression in the tumors in the combination therapy group (Figure 7E).

## 4. Discussion

CILP is a secretory protein mainly expressed in cartilage. It consists of two subtypes—*CILP1* and *CILP2*—which exhibit a 50.6% homology and differential expression in cartilage cells [27]. It has been suggested that CILP possesses the activity of nucleoside diphosphate kinase and plays an important role in carbohydrate binding and cartilage scaffolding [28]. *CILP1* can inhibit the response of insulin-like growth factor 1 (IGF-1) in chondrocytes, thereby impacting cell growth and repair, indirectly promoting hyperphosphatemia in aging and osteoarthritis, and increasing expression in early osteoarthritis patients [29,30,31,32,33]. *CILP2*, located on chromosome 19p13, is thought to play an important role in the progression of ankylosing spondylitis [34]. Additionally, it is elevated in atherosclerosis models and highly correlated with plasma lipid levels (e.g., HDL, LDL, TG, and TC), promoting macrophage lipid uptake and inducing foam cell formation [35]. Furthermore, *CILP2* is a susceptibility gene for diabetes and is closely related to the occurrence and development of diabetes [36]. Combining the above information, we found that *CILP2* plays a crucial regulatory role in the progression of chronic inflammation, which is crucial for promoting cancer occurrence and development through immunosuppression. Chronic inflammation is mainly related to immunosuppression, providing a favorable microenvironment for cancer occurrence, development, and metastasis. The inflammatory tumor microenvironment is a key determining factor for the effectiveness of cancer radiotherapy, chemotherapy, and immunotherapy. However, limited information is available regarding the role of *CILP2* in cancers, with only one study reporting that it may have served as a prognostic marker for colorectal cancer [8,9].

In our study, we evaluated the pan-cancer expression level of *CILP2* and found that it was significantly upregulated in most cancers. The analysis of the TCGA WGS/WES data revealed pan-cancer genetic alteration changes in *CILP2*, with most changes being mutations, and skin cutaneous melanoma having the most genetic variation, mainly in the form of mutations. CNV is a major contributor to genomic structural variation, impacting the expression of protein-coding and non-coding genes and the activities of various signaling pathways. The CNV analysis showed that the frequency of the copy number alterations in the *CILP2* gene was highly heterogeneous, with significant increases in UCEC, ESCA, and LIHC and significant decreases in LGG and TGCT. Meanwhile, in most cancers, the methylation of *CILP2* has been negatively correlated with its transcriptional levels. Therefore, the abnormal methylation and CNV of *CILP2* have led to poor prognoses in various cancers, indicating that the epigenetic changes in *CILP2* may promote the progression of certain cancers. The survival analysis demonstrated that *CILP2* was associated with the OS, PFI, DFI, and DSS in various cancers, especially with poor prognoses in ACC and KIRC. Furthermore, the univariate Cox regression analysis revealed that *CILP2* could serve as an independent prognostic risk factor for ACC, KIRC, and PAAD. These results indicated the importance of *CILP2* in the tumorigenesis and prognoses of cancers.

The tumor microenvironment (including immune components) is now regarded as a participant in tumorigenesis and progression, rather than as a bystander. In line with this notion, we evaluated the effects of *CILP2* on the microenvironment across 33 cancers at 3 levels, which showed that (1) at the tissue level, high expression levels of *CILP2* were consistently accompanied by lower tumor purities and more stroma; (2) at the cellular level, a more precise analysis of the various infiltrating cells in the tumor microenvironment indicated that *CILP2* was associated with TAM and CAF infiltration; and (3) at the molecular level, although *CILP2* exhibits heterogeneity in its association with most immunosuppressive genes across different cancers, *TGFB*/*TGFBR* consistently showed a positive correlation with *CILP2* in all of the examined cancers. Considering that MSI and TMB are currently identified as sensitive indicators for ICB in various cancers, we also analyzed the correlations between *CILP2* and MSI or TMB. The correlations between *CILP2* and TMB differed in the various cancers, while *CILP2* was negatively correlated with STAD, COAD, and CHOL. Coincidentally, the characteristics of pancreatic cancer are extremely consistent with the three levels of evaluation mentioned above. Pancreatic cancer has distinctive histopathological features whose TME presents a “cold” or “immune-excluded” landscape that is unable to initiate a strong immune response and poses a challenge to effective immunotherapy. This is also the reason why the five-year survival rate for pancreatic cancer is only 11%, and it is expected to become the second leading cause of death from malignant tumors in the United States by 2030. Combining our results with previous research results, we found that *CILP2* is highly expressed in pancreatic cancer tissues and increases the relative risk of pancreatic cancer, implying that *CILP2* may play an important role in pancreatic cancer.

Given the value of *CILP2* in pancreatic cancer that has been revealed by previous analyses, we then investigated its role using public databases and our clinical samples. The expression of *CILP2* has been consistently high in pancreatic cancer, as suggested by bioinformatic analyses, PCR, and IHC at multiple levels. Moreover, the subgroup analysis indicated higher expression levels of *CILP2* in the more aggressive (quasi-mesenchymal/basal/squamous) subtypes of pancreatic cancer, accompanied by poorer prognoses. The knockdown of *CILP2* in vitro restrained the proliferation, invasion, migration, and EMT of pancreatic cancer cells, which could be rescued by activating the AKT pathway. The overexpression of *CILP2* showed the opposite effect, and this was further validated using the orthotopic xenograft mouse model in vivo.

Considering the potential immune effect of *CILP2*, as indicated by the immune infiltration analysis, we also assessed a combination of the *CILP2* knockdown and immune checkpoint inhibitors in a mouse model. The result showed that the combination was superior compared with the treatment of the *CILP2* knockdown or immune checkpoint inhibitors alone. Further investigation at the pharmacologic level is currently limited by the absence of therapeutic *CILP2* antibodies or inhibitors. However, our results demonstrated the availability and efficiency of *CILP2* as a potential target for drug development, and our results also suggested that *CILP2* could serve as a therapeutic immune target for synergies with ICIs.

## 5. Conclusions

In summary, our study results indicated that *CILP2* is a potential tumor prognostic biomarker and immunotherapy target. We found that *CILP2* is significantly upregulated in most tumors, and its abnormal methylation and copy number variations are associated with poor prognoses in various cancers. Additionally, *CILP2* can serve as an independent prognostic risk factor for several cancers. In pancreatic cancer, high expression levels of *CILP2* lead to decreased tumor purities and increased stromal contents, which are closely related to TAM and CAF infiltration and strongly positively correlated with the expression of immune-suppressive genes. Finally, through in vitro and in vivo experiments, we confirmed that *CILP2* can regulate the proliferation, invasion, migration, and EMT of pancreatic cancer cells. Our study results will help to understand the role of *CILP2* in pan-cancer tumorigenesis and progression, especially in pancreatic cancer, and they provide the basis for further immunotherapy research.

## Figures and Tables

**Table 1:** The differential *CILP2* expression levels showed the relevance of the molecular subtypes in PDAC.

	Low (N = 75)	High (N = 75)	*p*
Subtype (Moffitt et al.):			0.003
Basal	23 (30.7%)	42 (56.0%)	
Classical	52 (69.3%)	33 (44.0%)	
Subtype (Collison et al.):			<0.001
QM(Quasi-mesenchymal)	7 (9.33%)	27 (36.0%)	
Classical	28 (37.3%)	26 (34.7%)	
Exocrine	40 (53.3%)	22 (29.3%)	
Subtype (Bailey et al.):			<0.001
Squamous	10 (13.3%)	21 (28.0%)	
Progenitor	36 (48.0%)	17 (22.7%)	
Immunogenic	5 (6.67%)	23 (30.7%)	
ADEX	24 (32.0%)	14 (18.7%)	

## Data Availability

The data from the TCGA and GEO datasets used in this study are publicly available.

## Supplementary Materials

The following supporting information can be downloaded at: https://www.mdpi.com/article/10.3390/cancers15245842/s1, Table S1: The sequences of the siRNA (5′-3′). Table S2: The sequences of the qRT-PCR primers. Table S3: The antibodies. Table S4: The clinicopathologic characteristics of *CILP2* low vs. high in PDAC. Figure S1: (A–G) Higher methylation levels of *CILP2* were associated with poorer OS in GBM, LGG, LIHC, PAAD, SARC, STAD, and UVM. (H) Higher methylation levels of *CILP2* were associated with poorer PFI in ESCA. (I) Higher methylation levels of *CILP2* were associated with poorer DFI in ESCA. (J) Higher methylation levels of *CILP2* were associated with poorer DSS in UVM. Figure S2: *CILP2* expression levels are strongly associated with the infiltration of CAFs and TAMs in pan-cancer (TIMER database). E. (A) *CILP2* expression in the BxPC-3 cells after transfection with the siRNA-CILP2s or the si-NC was detected by qRT-PCR. (B) The *CILP2* expression in PANC-1 after transfection with pcDNA3.1-NC or pcDNA3.1-CILP2 was detected by qRT-PCR. (C,D) The effects of *CILP2* overexpression on proliferation in the PANC-1 cells were measured by the CCK-8 and colony formation assays. (E,F) The effects of *CILP2* overexpression on proliferation in the PANC-1 cells were measured by the transwell and wound healing assays. (G) The effects of *CILP2* overexpression on EMT and the PI3K-AKT pathway in the PANC-1 cells were measured by Western blot. Figure S3: (A) CILP2 expression in BxPC-3 cells after transfection with siRNA-CILP2s or si-NC was detected by qRT-PCR. (B) CILP2 expression in PANC-1 after transfection with pcDNA3.1-NC or pcDNA3.1-CILP2 was detected by qRT-PCR. (C,D) The effects of CILP2 overexpression on proliferation in PANC-1 cells were measured by CCK-8 assay and colony formation assay. (E,F) The effects of CILP2 overexpression on proliferation in PANC-1 cells were measured by Transwell assay and wound healing assay. (G) The effects of CILP2 overexpression on EMT and PI3K-AKT pathway in PANC-1 cells were measured by western blot. Figure S4: (A,B) The CCK-8 assay and colony formation assay, respectively, were carried out to examine cell proliferation in the PANC-1 cells transfected with pcDNA3.1-CILP2 and treated with LY294002 or the corresponding negative control. (C,D) The transwell assay and wound healing assay, respectively, were used to examine the cell migration and invasion of the PANC-1 cells transfected with pcDNA3.1-CILP2 and treated with LY294002 or the corresponding negative control. (E) The Western blot assay was performed to examine the expression of EMT in the PANC-1 cells transfected with pcDNA3.1-CILP2 and treated with LY294002 or the corresponding negative control. Figure S5: (A) The IHC staining assay was performed to examine the expression of Ki-67, E-cadherin, N-cadherin, and vimentin. (B) The *CILP2* expression in the Panc02 cells after transfection with the siRNA-CILP2-mouse or the si-NC-mouse was detected by qRT-PCR. File S1: Original western blots.

## Abbreviations

LAML, acute myeloid leukemia; ACC, adrenocortical carcinoma; BLCA, bladder urothelial carcinoma; LGG, brain lower-grade glioma; BRCA, breast-invasive carcinoma; CESC, cervical squamous cell carcinoma and endocervical adenocarcinoma; CHOL, cholangiocarcinoma; COAD, colon adenocarcinoma; DLBC, diffuse large B-cell lymphoma; ESCA, esophageal carcinoma; GBM, glioblastoma multiforme; HNSC, head and neck squamous cell carcinoma; KICH, kidney chromophobe; KIRC, kidney renal clear cell carcinoma; KIRP, kidney renal papillary cell carcinoma; LIHC, liver hepatocellular carcinoma; LUAD, lung adenocarcinoma; LUSC, lung squamous cell carcinoma; MESO, mesothelioma; OV, ovarian serous cystadenocarcinoma; PAAD, pancreatic adenocarcinoma; PCPG, pheochromocytoma and paraganglioma; PRAD, prostate adenocarcinoma; READ, rectum adenocarcinoma; SARC, sarcoma; SKCM, skin cutaneous melanoma; STAD, stomach adenocarcinoma; STES, stomach and esophageal carcinoma; TGCT, testicular germ cell tumors; THCA, thyroid carcinoma; THYM, thymoma; UCS, uterine carcinosarcoma; UCEC, uterine corpus endometrial carcinoma; UVM, uveal melanoma; CILP2, cartilage intermediate layer protein 2; TGFB, transforming growth factor beta; TGFBR, transforming growth factor beta receptor; EMT, epithelial–mesenchymal transition; CNV, copy number variation; PDAC, pancreatic ductal adenocarcinoma; ECM, extracellular matrix; GSVA, gene set variation analysis; CCK-8, cell counting kit 8; IHC, immunohistochemistry; ICIs, immune checkpoint inhibitors.

## References

[1] Siegel R.L., Miller K.D., Wagle N.S., Jemal A. (2023). Cancer statistics, 2023. CA Cancer J. Clin..

[2] Sung H., Ferlay J., Siegel R.L., Torre L.A., Jemal A. (2021). Global Cancer Statistics 2020: GLOBOCAN Estimates of Incidence and Mortality Worldwide for 36 Cancers in 185 Countries. CA Cancer J. Clin..

[3] Wahida A., Buschhorn L., Fröhling S., Jost P.J., Schneeweiss A., Lichter P., Kurzrock R. (2023). The coming decade in precision oncology: Six riddles. Nat. Rev. Cancer.

[4] Bedard P.L., Hyman D.M., Davids M.S., Siu L.L. (2020). Small molecules, big impact: 20 years of targeted therapy in oncology. Lancet.

[5] Jiang P., Sinha S., Aldape K., Hannenhalli S., Sahinalp C., Ruppin E. (2022). Big data in basic and translational cancer research. Nat. Rev. Cancer.

[6] Bernardo B.C., Belluoccio D., Rowley L., Little C.B., Hansen U., Bateman J.F. (2011). Cartilage intermediate layer protein 2 (CILP-2) is expressed in articular and meniscal cartilage and down-regulated in experimental osteoarthritis. J. Biol. Chem..

[7] Zhang C.L., Zhao Q., Liang H., Qiao X., Wang J.Y., Wu D., Wu L.L., Li L. (2018). Cartilage intermediate layer protein-1 alleviates pressure overload-induced cardiac fibrosis via interfering TGF-β1 signaling. J. Mol. Cell. Cardiol..

[8] Huang F., Peng Y., Ye Q., Chen J., Li Y., Liu S., Xu Y., Huang L. (2020). CILP2 overexpression correlates with tumor progression and poor prognosis in patients with colorectal cancer in The Cancer Genome Atlas (TCGA) study. World J. Surg. Oncol..

[9] Wang X., Zhang Y., Song N., Li K., Lei S., Wang J., Wang Z., Zhang W. (2023). CILP2: A prognostic biomarker associated with immune infiltration in colorectal cancer. Heliyon.

[10] Barretina J., Caponigro G., Stransky N., Venkatesan K., Margolin A.A., Kim S., Wilson C.J., Lehár J., Kryukov G.V., Sonkin D. (2012). The Cancer Cell Line Encyclopedia enables predictive modelling of anticancer drug sensitivity. Nature.

[11] The Cancer Genome Atlas Research Network (2017). Integrated Genomic Characterization of Pancreatic Ductal Adenocarcinoma. Cancer Cell.

[12] Collisson E.A., Sadanandam A., Olson P., Gibb W.J., Truitt M., Gu S., Cooc J., Weinkle J., Kim G.E., Jakkula L. (2011). Subtypes of pancreatic ductal adenocarcinoma and their differing responses to therapy. Nat. Med..

[13] Moffitt R.A., Marayati R., Flate E.L., Volmar K.E., Loeza S.G.H., Hoadley K.A., Rashid N.U., Williams L.A., Eaton S.C., Chung A.H. (2015). Virtual microdissection identifies distinct tumor- and stroma-specific subtypes of pancreatic ductal adenocarcinoma. Nat. Genet..

[14] Bailey P., Chang D.K., Nones K., Johns A.L., Patch A.M., Gingras M.C., Miller D.K., Christ A.N., Bruxner T.J., Quinn M.C. (2016). Genomic analyses identify molecular subtypes of pancreatic cancer. Nature.

[15] Cerami E., Gao J., Dogrusoz U., Gross B.E., Sumer S.O., Aksoy B.A., Jacobsen A., Byrne C.J., Heuer M.L., Larsson E. (2012). The cBio cancer genomics portal: An open platform for exploring multidimensional cancer genomics data. Cancer Discov..

[16] Gao J., Aksoy B.A., Dogrusoz U., Dresdner G., Gross B., Sumer S.O., Sun Y., Jacobsen A., Sinha R., Larsson E. (2013). Integrative analysis of complex cancer genomics and clinical profiles using the cBioPortal. Sci. Signal..

[17] Wu T., Hu E., Xu S., Chen M., Guo P., Dai Z., Feng T., Zhou L., Tang W., Zhan L.I. (2021). clusterProfiler 4.0: A universal enrichment tool for interpreting omics data. Innov. Camb. Mass..

[18] Subramanian A., Tamayo P., Mootha V.K., Mukherjee S., Ebert B.L., Gillette M.A., Paulovich A., Pomeroy S.L., Golub T.R., Lander E.S. (2005). Gene set enrichment analysis: A knowledge-based approach for interpreting genome-wide expression profiles. Proc. Natl. Acad. Sci. USA.

[19] Gene Ontology Consortium (2015). Gene Ontology Consortium: Going forward. Nucleic Acids Res..

[20] Kanehisa M., Furumichi M., Tanabe M., Sato Y., Morishima K. (2017). KEGG: New perspectives on genomes, pathways, diseases and drugs. Nucleic Acids Res..

[21] Liberzon A., Birger C., Thorvaldsdóttir H., Ghandi M., Mesirov J.P., Tamayo P. (2015). The Molecular Signatures Database (MSigDB) hallmark gene set collection. Cell Syst..

[22] Yoshihara K., Shahmoradgoli M., Martínez E., Vegesna R., Kim H., Torres-Garcia W., Treviño V., Shen H., Laird P.W., Levine D.A. (2013). Inferring tumour purity and stromal and immune cell admixture from expression data. Nat. Commun..

[23] Chen B., Khodadoust M.S., Liu C.L., Newman A.M., Alizadeh A.A. (2018). Profiling Tumor Infiltrating Immune Cells with CIBERSORT. Cancer Syst. Biol. Methods Protoc..

[24] Cheng C., Liu D., Liu Z., Li M., Wang Y., Sun B., Kong R., Chen H., Wang G., Li L. (2022). Positive feedback regulation of lncRNA TPT1-AS1 and ITGB3 promotes cell growth and metastasis in pancreatic cancer. Cancer Sci..

[25] Geng X., Li L., Luo Y., Yang W., Hu J., Zhao Z., Cheng C., Zhang T., Zhang Y., Liu L. (2023). Tumor Cell Derived Lnc-FSD2-31:1 Contributes to Cancer-Associated Fibroblasts Activation in Pancreatic Ductal Adenocarcinoma Progression through Extracellular Vesicles Cargo MiR-4736. Adv. Sci..

[26] Collisson E.A., Bailey P., Chang D.K., Biankin A.V. (2019). Molecular subtypes of pancreatic cancer. Nat. Rev. Gastroenterol. Hepatol..

[27] Raggatt L.J., Partridge N.C. (2010). Cellular and molecular mechanisms of bone remodeling. J. Biol. Chem..

[28] Dehne T., Karlsson C., Ringe J., Sittinger M., Lindahl A. (2009). Chondrogenic differentiation potential of osteoarthritic chondrocytes and their possible use in matrix-associated autologous chondrocyte transplantation. Arthritis Res. Ther..

[29] Johnell O., Kanis J.A. (2006). An estimate of the worldwide prevalence and disability associated with osteoporotic fractures. Osteoporos. Int..

[30] Feng X., McDonald J.M. (2011). Disorders of bone remodeling. Annu. Rev. Pathol..

[31] Pazianas M., Miller P.D. (2021). Osteoporosis and Chronic Kidney Disease-Mineral and Bone Disorder (CKD-MBD): Back to Basics. Am. J. Kidney Dis..

[32] Marahleh A., Kitaura H., Ohori F., Noguchi T., Mizoguchi I. (2023). The osteocyte and its osteoclastogenic potential. Front. Endocrinol..

[33] Deng G.X., Yin R.X., Guan Y.Z., Liu C.X., Zheng P.F., Wei B.L., Wu J.Z., Miao L. (2020). Association of the NCAN-TM6SF2-CILP2-PBX4-SUGP1-MAU2 SNPs and gene-gene and gene-environment interactions with serum lipid levels. Aging.

[34] Carlberg K., Korotkova M., Larsson L., Catrina A.I., Ståhl P.L., Malmström V. (2019). Exploring inflammatory signatures in arthritic joint biopsies with Spatial Transcriptomics. Sci. Rep..

[35] Hu W., Li K., Han H., Geng S., Zhou B., Fan X., Xu S., Yang M., Liu H., Yang G. (2020). Circulating Levels of CILP2 Are Elevated in Coronary Heart Disease and Associated with Atherosclerosis. Oxid. Med. Cell. Longev..

[36] Wu T., Zhang Q., Wu S., Hu W., Zhou T., Li K., Liu D., Gu H.F., Zheng H., Zhu Z. (2019). CILP-2 is a novel secreted protein and associated with insulin resistance. J. Mol. Cell Biol..

