# Supplementary material for: The Prognostic and Immune Significance of CILP2 in Pan-Cancer and Its Relationship with the Progression of Pancreatic Cancer
Source: Cancers (Basel). 2023 Dec 14;15(24):5842. doi: 10.3390/cancers15245842 (PMC10741840; doi:10.3390/cancers15245842)
Supplement: Supplementary file 1 [file cancers-15-05842-s001.zip › File S1-Full length western blots.pptx]

## Slide 1
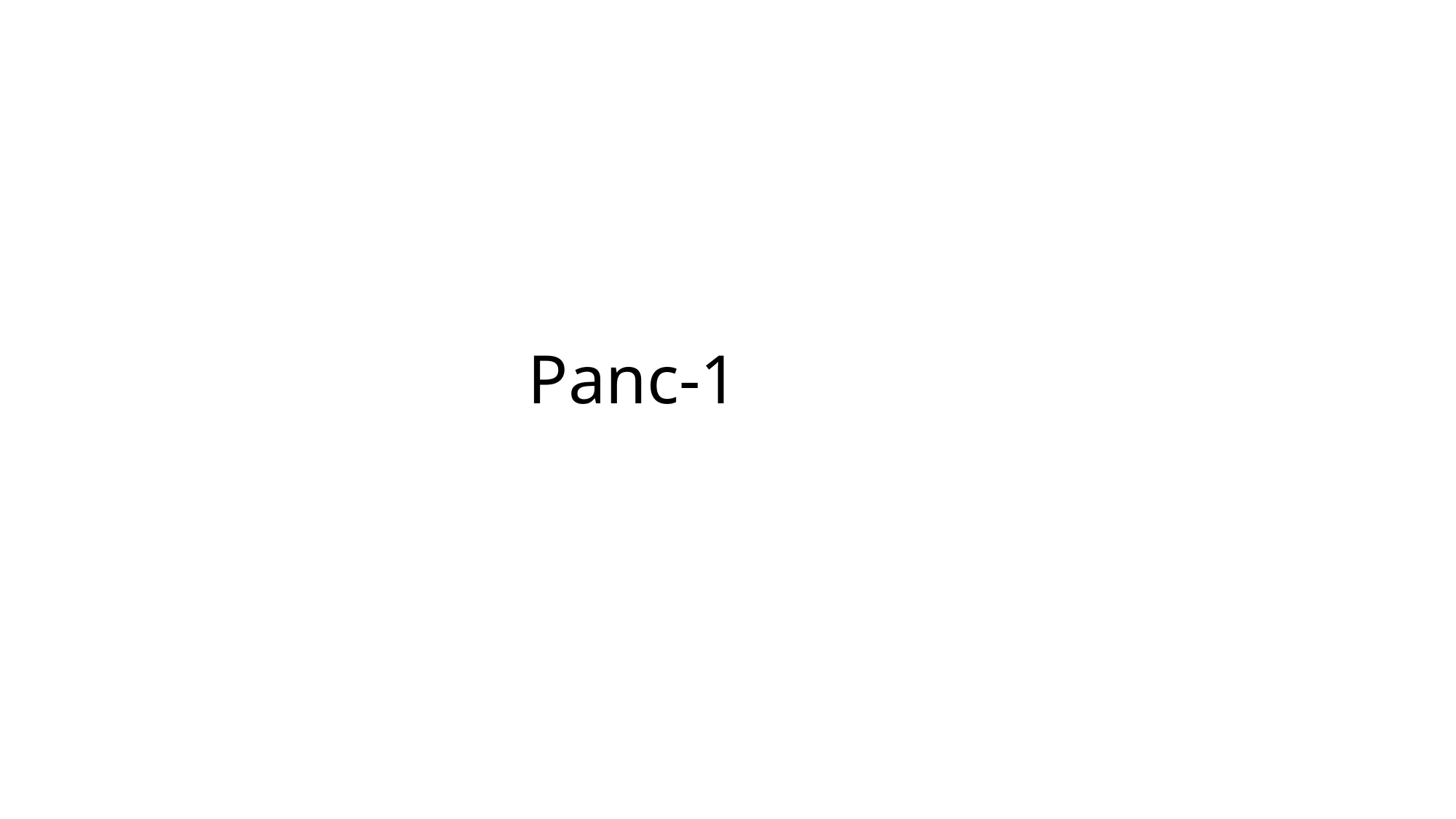

# Panc-1

## Slide 2
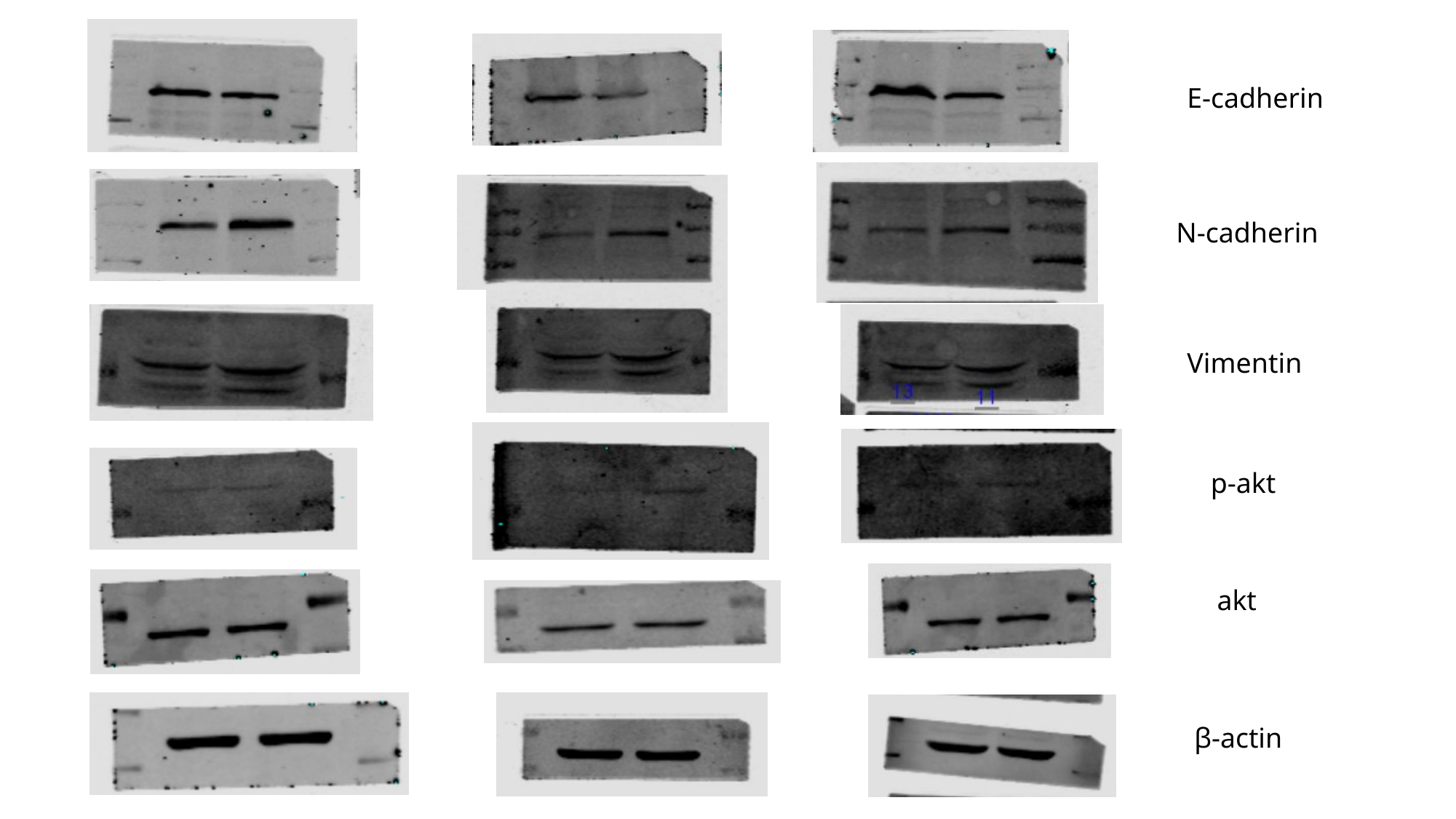

E-cadherin
N-cadherin
Vimentin
p-akt
akt
β-actin

## Slide 3
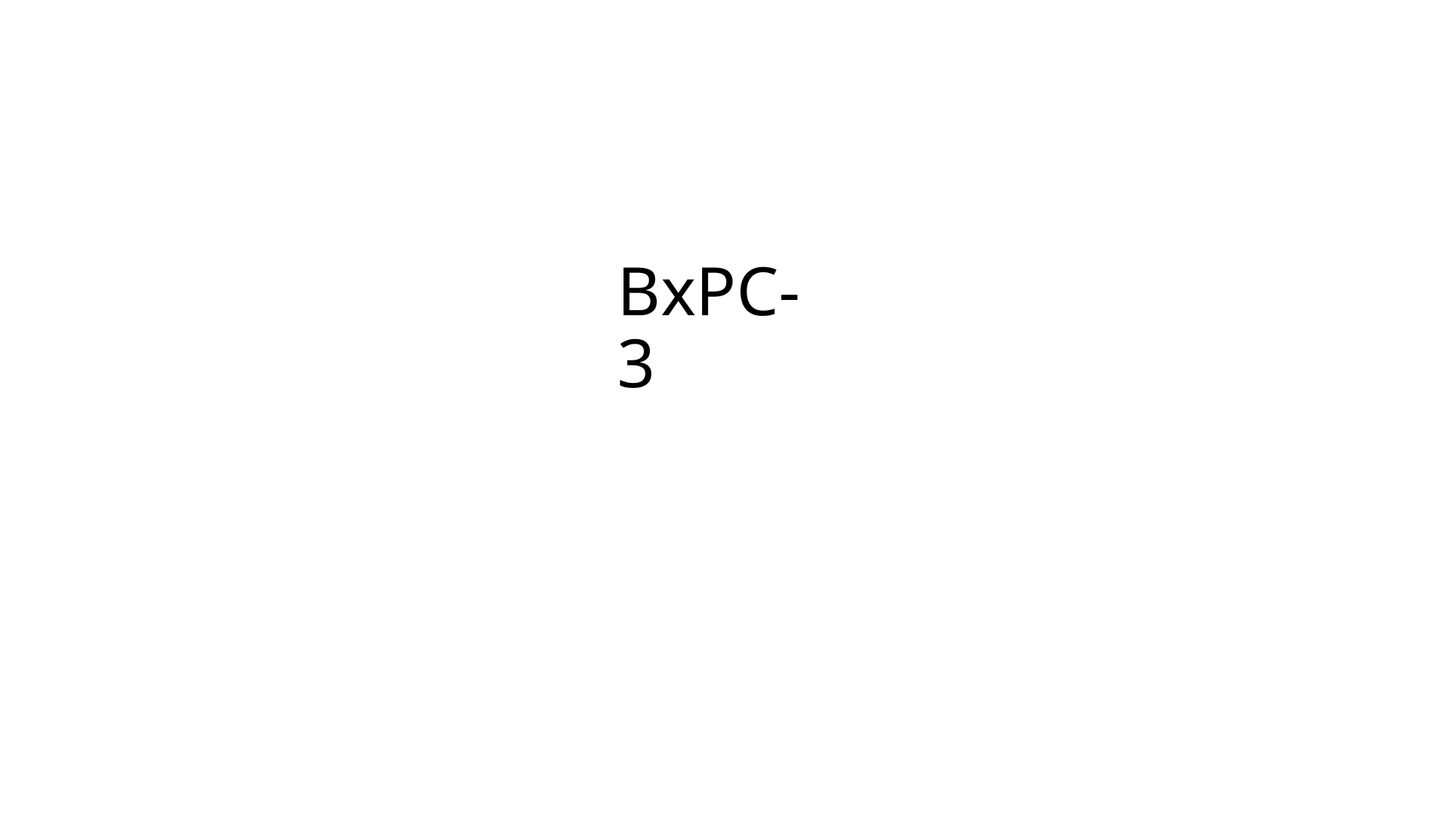

# BxPC-3

## Slide 4
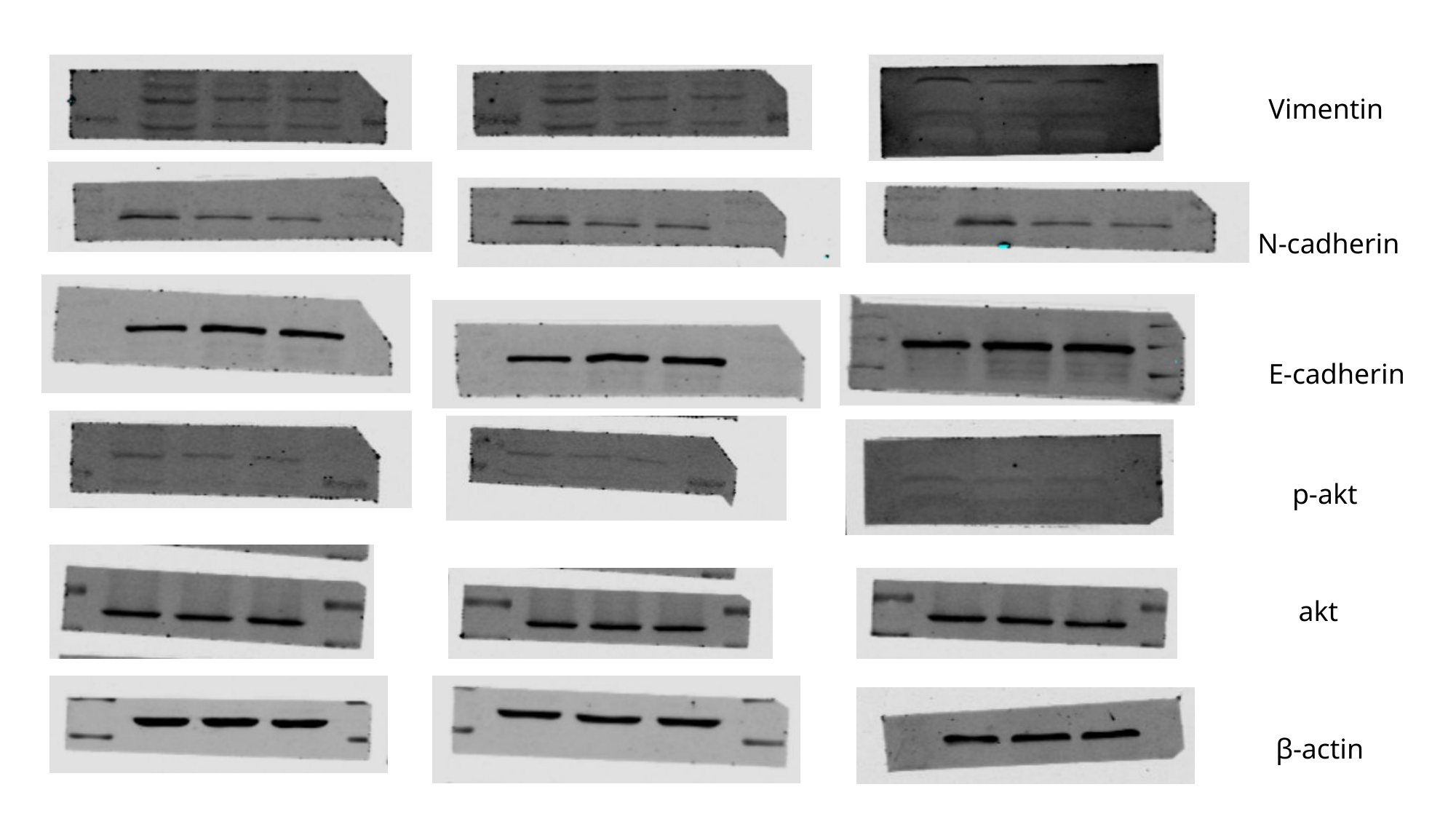

Vimentin
N-cadherin
E-cadherin
p-akt
akt
β-actin
