# Supplementary material for: The Prognostic and Immune Significance of CILP2 in Pan-Cancer and Its Relationship with the Progression of Pancreatic Cancer
Source: Cancers (Basel). 2023 Dec 14;15(24):5842. doi: 10.3390/cancers15245842 (PMC10741840; doi:10.3390/cancers15245842)
Supplement: Supplementary file 1 [file cancers-15-05842-s001.zip › Table S1. The sequences of siRNA (5í»-3í»).pdf]

Table S1. The sequences of siRNA (5’-3’).

siRNA name	Sequence
si-CILP2-1	GCGAUGCUCACGGAACCUUTT<br>AAGGUUCCGUGAGCAUCGCTT
si-CILP2-2	CCAACUACCACGUGCGCUUTT<br>AAGCGCACGUGGUAGUUGGTT
si-CILP2-3	GCUACGCUUCGCCAGGAUUTT<br>AAUCCUGGCGAAGCGUAGCTT
si-NC	UUCUCCGAACGUGUCACGUTT<br>ACGUGACACGUUCGGAGAATT
si-CILP2-1-mouse	ACCGAGGAGCACACUUCAACCTT<br>GGUUGAAGUGUGCUCCUCGGUTT
si-CILP2-2-mouse	CAUCAUCCUUGAAGAGUUAGGTT<br>CCUAACUCUUCAAGGAUGAUGTT
si-CILP2-3-mouse	CGACCUCAGGAACACCUGAUCTT<br>GAUCAGGUGUUCCUGAGGUCGTT
si-NC-mouse	UUCUCCGAACGUGUCACGUTT<br>ACGUGACACGUUCGGAGAATT
