# Supplementary material for: The Prognostic and Immune Significance of CILP2 in Pan-Cancer and Its Relationship with the Progression of Pancreatic Cancer
Source: Cancers (Basel). 2023 Dec 14;15(24):5842. doi: 10.3390/cancers15245842 (PMC10741840; doi:10.3390/cancers15245842)
Supplement: Supplementary file 1 [file cancers-15-05842-s001.zip › Table S2. The sequences of the qRT-PCR primers.pdf]

**Table S2. The sequences of the qRT-PCR primers.**

| Gene | Species | Forward primer |
|---|---|---|
| *CILP2* | human | F: GCAAGGCATGGAATGAGGCGG |
| | | R: CACTACCTGGCTGACCACAGT C |
| *ACTB* | human | F: CACCATTGGCAATGAGCGGTTC |
| | | R: AGGTCTTTGCGGATGTCCACGT |
| *Cilp2* | Mouse | F: GATGAATGGGTTCTCTGCTGGC |
| | | R: TTCTGACCTGCCTCTCGAACTC |
| *Actb* | Mouse | F: CATTGCTGACAGGATGCAGAAGG |
| | | R: TGCTGGAAGGTGGACAGTGAGG |
