# Supplementary material for: The Prognostic and Immune Significance of CILP2 in Pan-Cancer and Its Relationship with the Progression of Pancreatic Cancer
Source: Cancers (Basel). 2023 Dec 14;15(24):5842. doi: 10.3390/cancers15245842 (PMC10741840; doi:10.3390/cancers15245842)
Supplement: Supplementary file 1 [file cancers-15-05842-s001.zip › Table S3. Antibodies.pdf]

Table S3. Antibodies

CILP2	Bioss	bs-13955R	IHC
E-cadherin	Cell Signaling Technology	#3195	WB, IHC
N-cadherin	Proteintech	22018-1-AP	WB, IHC
Vimentin	Proteintech	10366-1-AP	WB, IHC
AKT	Cell Signaling Technology	#4691	WB
p-AKT	Cell Signaling Technology	#4060	WB
Ki-67	Cell Signaling Technology	#62548	IHC
β-actin	ZSGB-BIO	TA-09	WB
