# Supplementary material for: The Prognostic and Immune Significance of CILP2 in Pan-Cancer and Its Relationship with the Progression of Pancreatic Cancer
Source: Cancers (Basel). 2023 Dec 14;15(24):5842. doi: 10.3390/cancers15245842 (PMC10741840; doi:10.3390/cancers15245842)
Supplement: Supplementary file 1 [file cancers-15-05842-s001.zip › Table S4 Clinicopathologic characteristic of CILP2 low vs. high in PDAC.pdf]

Table S4 Clinicopathologic characteristic of CILP2 low vs. high in PDAC

	Low(N=75)	High(N=75)	*P*
**Age:**			0.075
<60	17 (22.7%)	28 (37.3%)	
≥60	58 (77.3%)	47 (62.7%)	
**Gender:**			0.743
female	36 (48.0%)	33 (44.0%)	
male	39 (52.0%)	42 (56.0%)	
**Vital status:**			0.412
Alive	31 (41.3%)	37 (49.3%)	
Dead	44 (58.7%)	38 (50.7%)	
**Tumor location:**			0.816
Body/Tail	10 (14.9%)	12 (17.9%)	
Head	57 (85.1%)	55 (82.1%)	
**Grade:**			0.315
G1	4 (5.33%)	1 (1.33%)	
G2	39 (52.0%)	36 (48.0%)	
G3	32 (42.7%)	37 (49.3%)	
G4	0 (0.00%)	1 (1.33%)	
**Staging:**			0.595
I	5 (6.67%)	7 (9.59%)	
II	67 (89.3%)	62 (84.9%)	
III	2 (2.67%)	1 (1.37%)	
IV	1 (1.33%)	3 (4.11%)	
**T stage:**			1.000
T1	2 (2.67%)	3 (4.05%)	
T2	8 (10.7%)	8 (10.8%)	
T3	63 (84.0%)	62 (83.8%)	
T4	2 (2.67%)	1 (1.35%)	
**N stage:**			1.000
N0	20 (26.7%)	19 (25.3%)	
N1	55 (73.3%)	55 (73.3%)	
NX	0 (0.00%)	1 (1.33%)	
**M stage:**			0.525
M0	36 (48.0%)	32 (42.7%)	
M1	1 (1.33%)	3 (4.00%)	
MX	38 (50.7%)	40 (53.3%)	
