# Supplementary figures and images for: The Prognostic and Immune Significance of CILP2 in Pan-Cancer and Its Relationship with the Progression of Pancreatic Cancer
Source: Cancers (Basel). 2023 Dec 14;15(24):5842. doi: 10.3390/cancers15245842 (PMC10741840; doi:10.3390/cancers15245842)

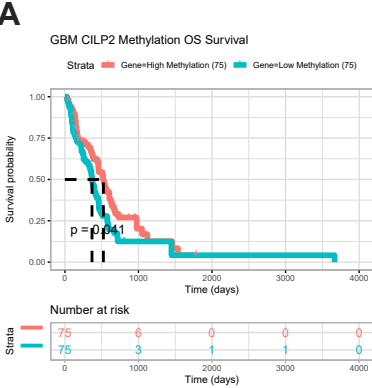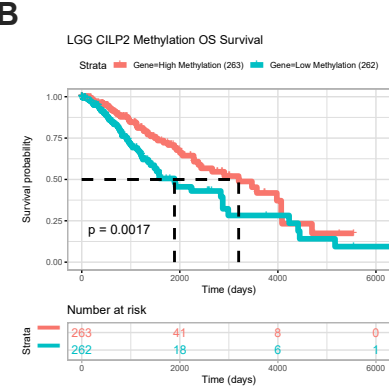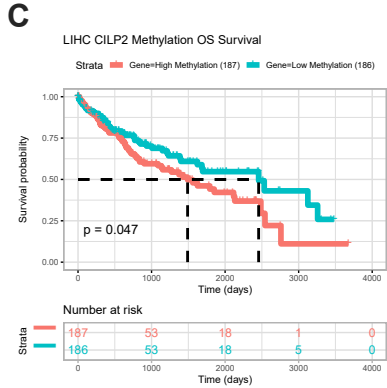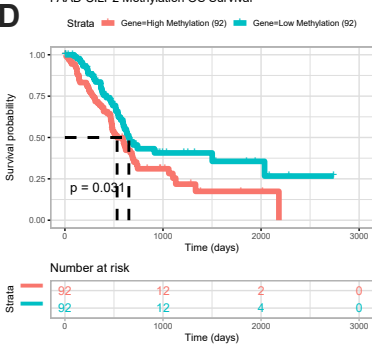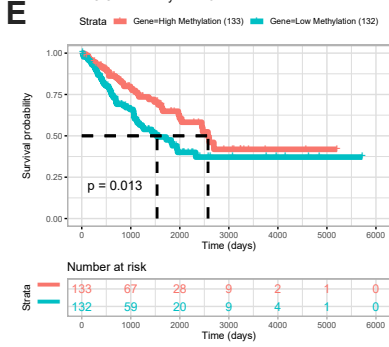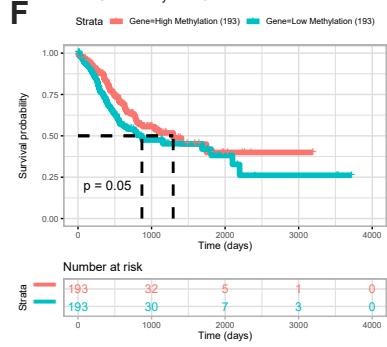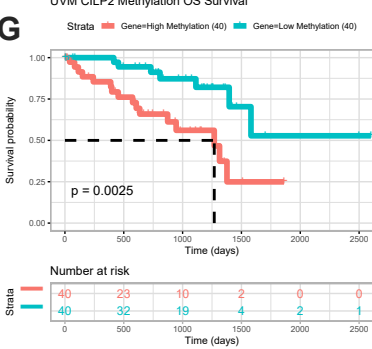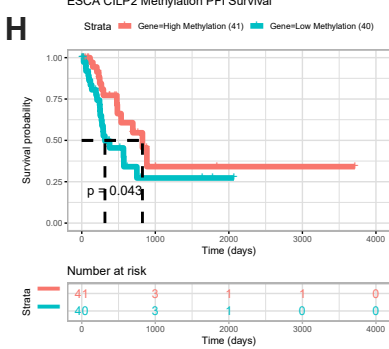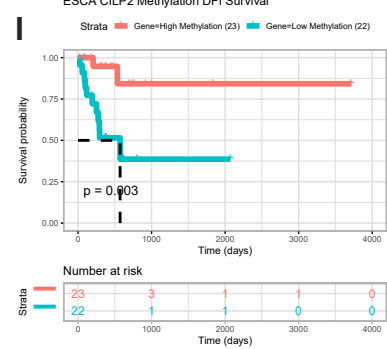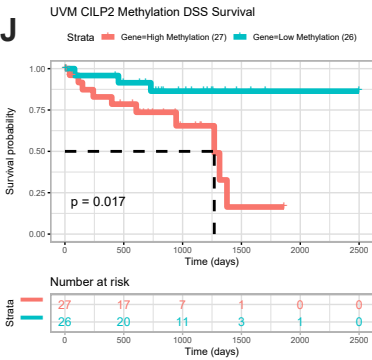

Supplement: Supplementary file 1 [file cancers-15-05842-s001.zip › Figure S1.pdf]

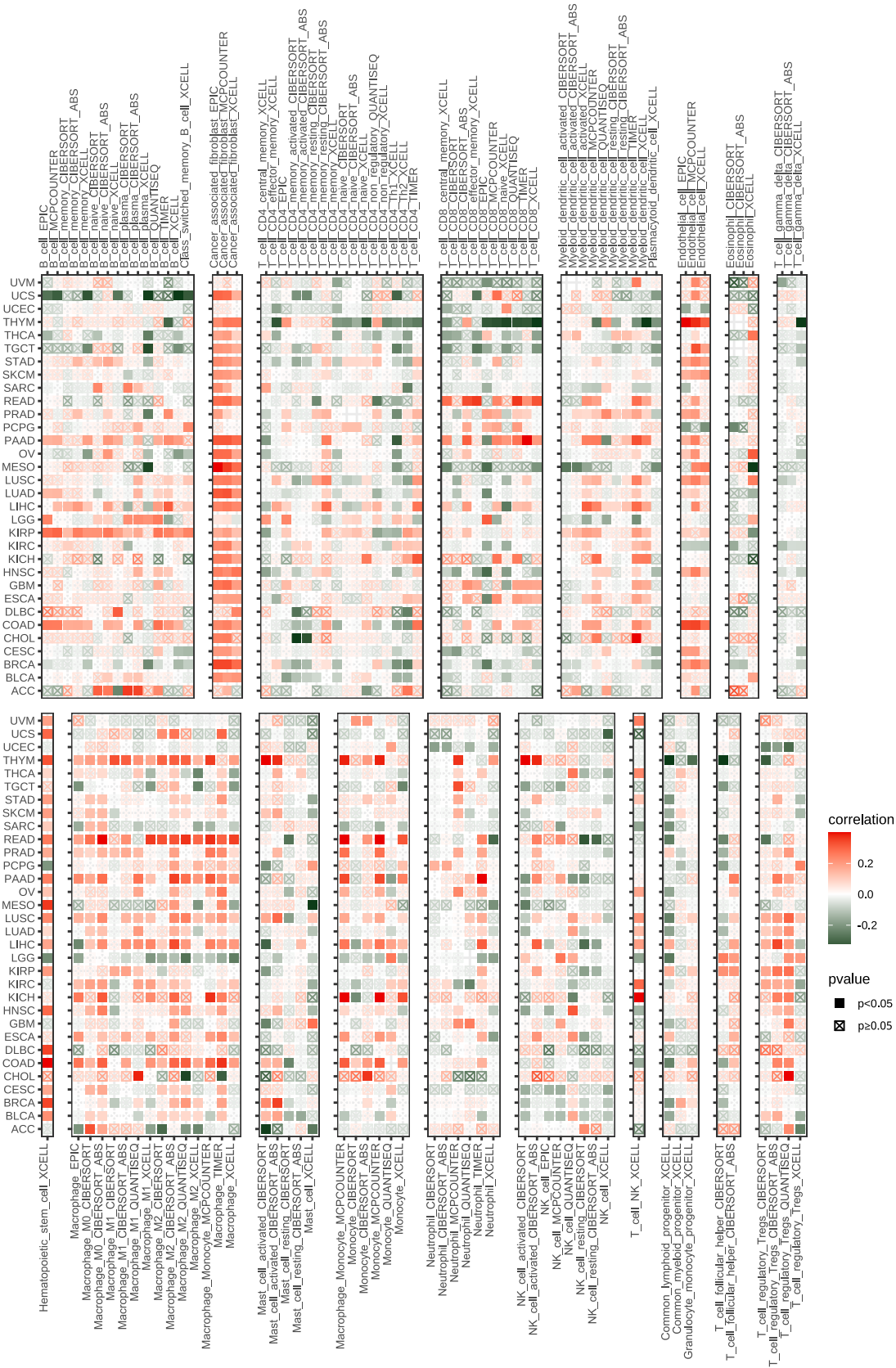

Supplement: Supplementary file 1 [file cancers-15-05842-s001.zip › Figure S2.pdf]

**A**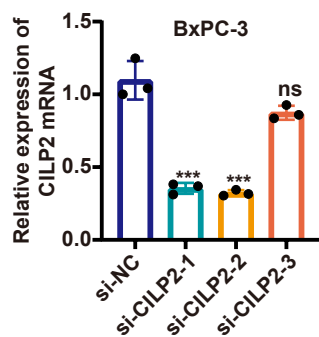**B**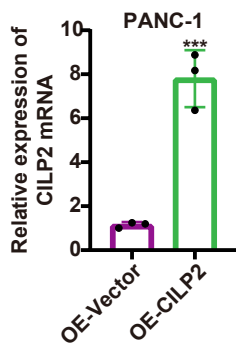**C**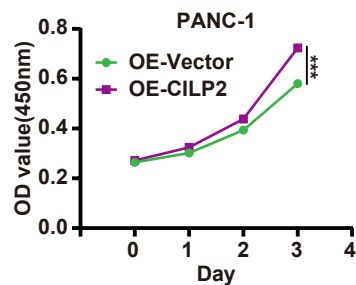**D**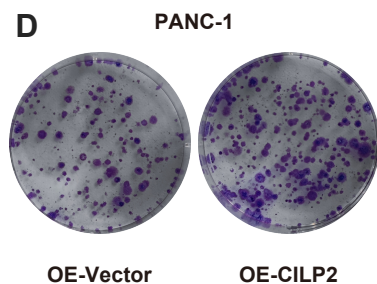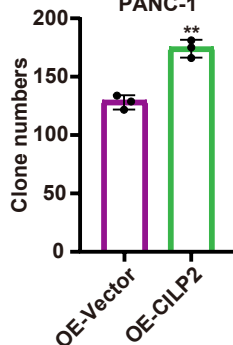**E**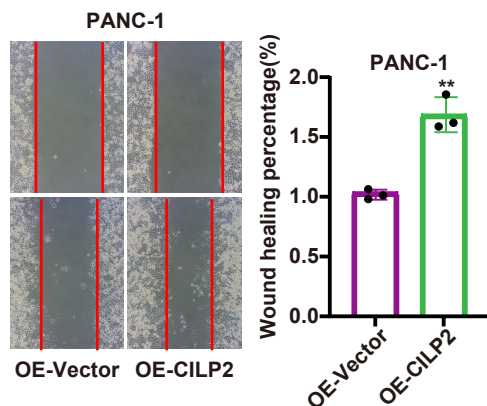**F**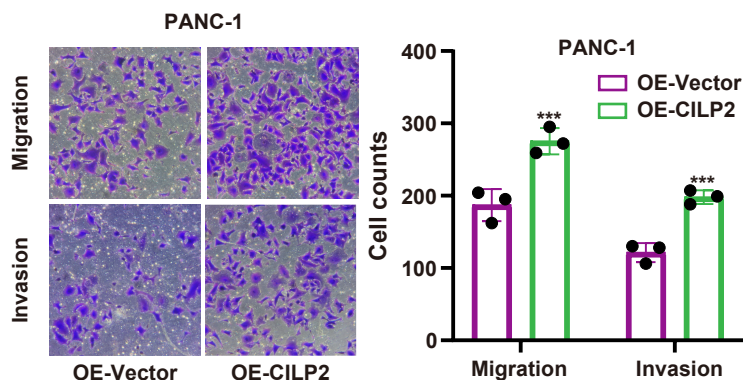**G**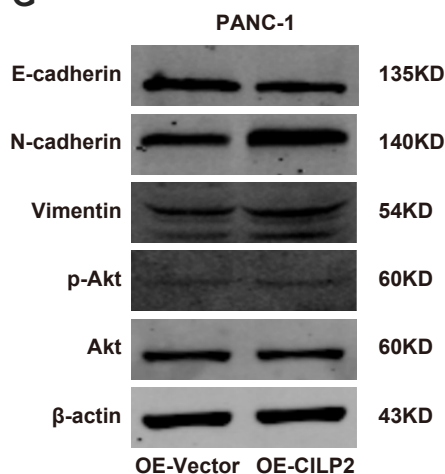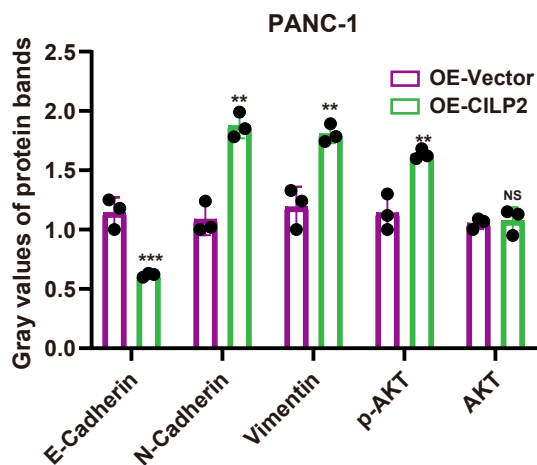

Supplement: Supplementary file 1 [file cancers-15-05842-s001.zip › Figure S3.pdf]

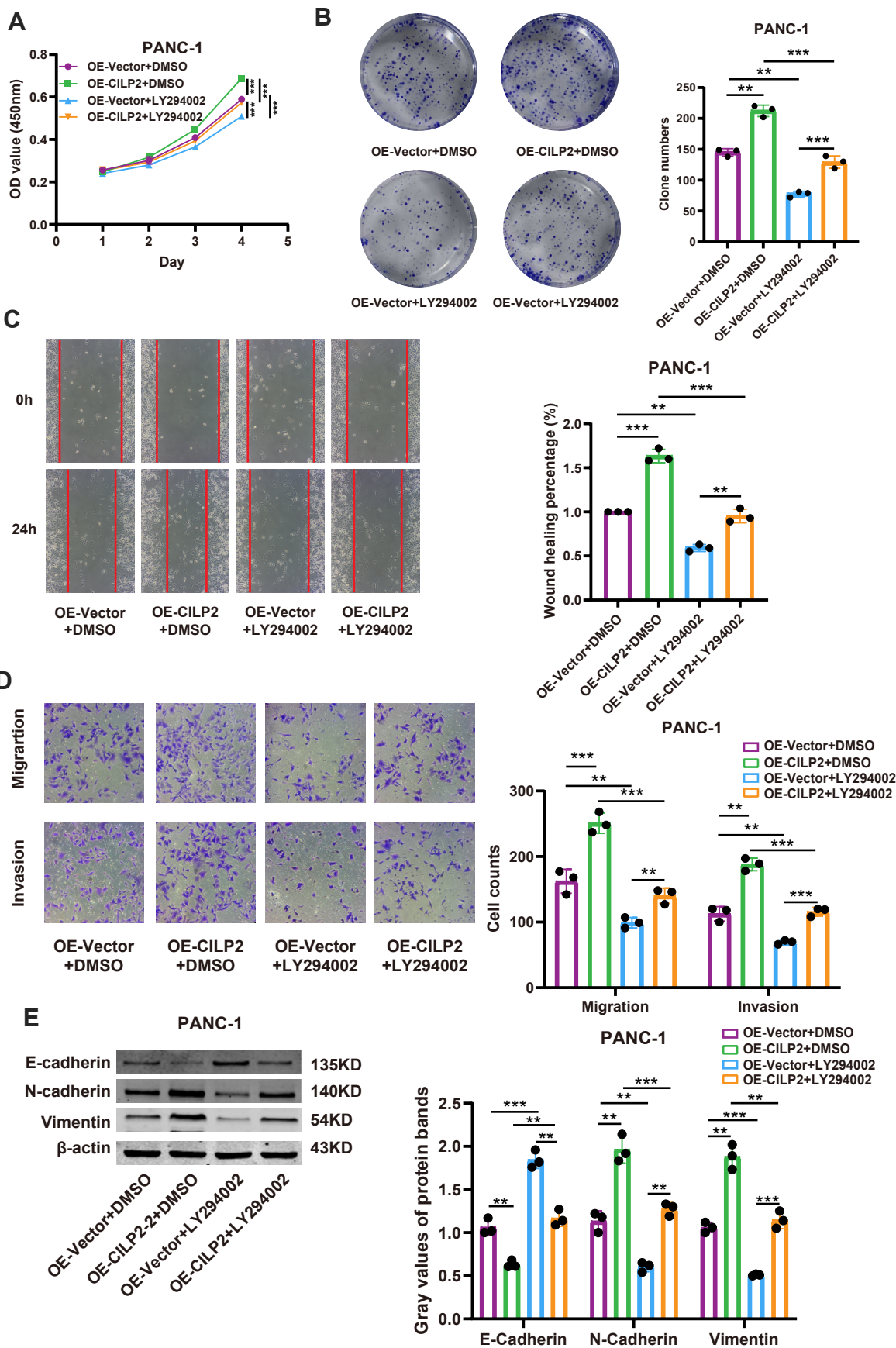

Supplement: Supplementary file 1 [file cancers-15-05842-s001.zip › Figure S4.pdf]

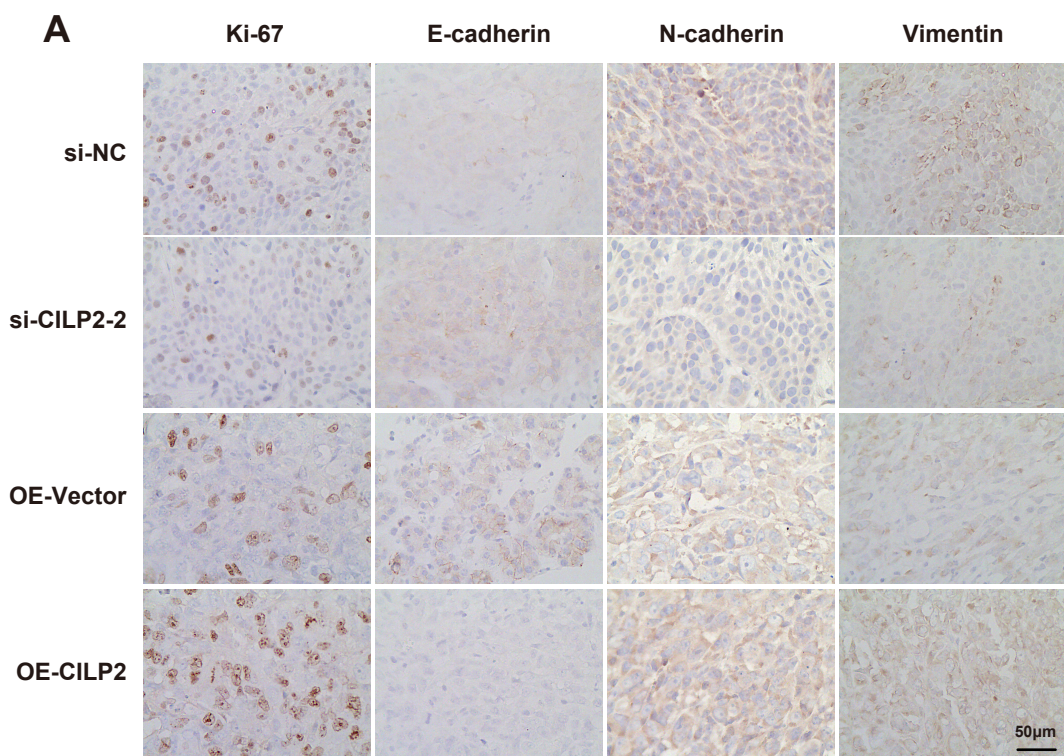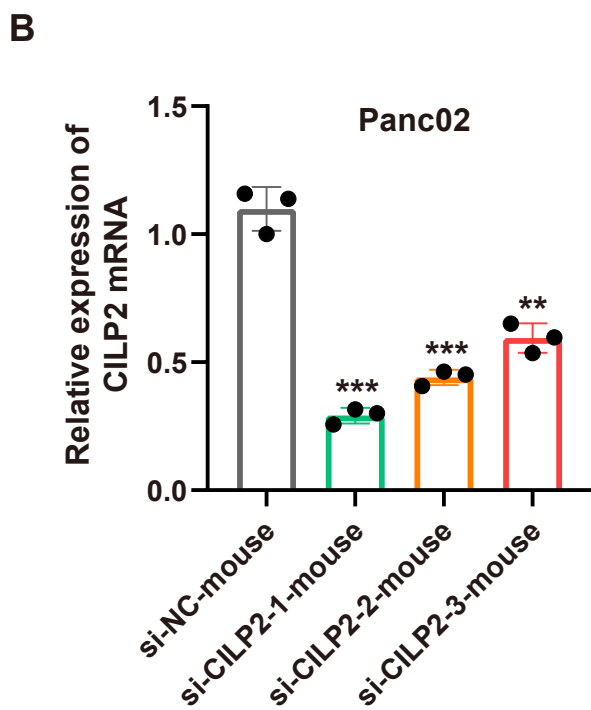

Supplement: Supplementary file 1 [file cancers-15-05842-s001.zip › Figure S5.pdf]
